# Supplementary material for: A meta-learning approach for selectivity prediction in asymmetric catalysis
Source: Nat Commun. 2025 Apr 16;16:3599. doi: 10.1038/s41467-025-58854-8 (PMC12000603; doi:10.1038/s41467-025-58854-8)
Supplement: Supplementary file 1 — Supplementary Information [file 41467_2025_58854_MOESM1_ESM.pdf]

Supplementary Information for

A Meta-learning Approach for Selectivity Prediction in Asymmetric Catalysis

Sukriti Singh* and José Miguel Hernández-Lobato*

Department of Engineering, University of Cambridge, Cambridge CB2 1PZ, U.K.

sukriti243@gmail.com; jmh233@cam.ac.uk

Section	Table of Contents	Page No.
1.	Implementation	S3
	1.1 Single-task methods	S3
	1.2 Meta-learning methods	S3
2.	Model performance with fingerprint-based input representation	S4
	2.1 Performance of meta-learning methods	S4
	2.2 Performance of single-task methods	S5
3.	Model performance with graph-based input representation	S6
	3.1 Performance of meta-learning methods	S6
	3.2 Performance of single-task methods	S7
4.	Dataset preparation for meta-cluster approach	S8
5.	Model performance with meta-cluster approach	S10
	5.1 Performance of meta-learning methods	S10
	5.2 Performance of single-task methods	S12
6.	Model performance with different classification thresholds	S15
7.	Identity of predictions	S16
8.	Effect of clustering on performance of meta-cluster model	S45
9.	Cluster-based train-test tasks for meta-learning	S46
10.	Performance on out-of-sample test set	S49

1. Implementation

In this section, we describe the implementation details of meta-learning and single-task methods with fingerprint and graph representations.

1.1 Single-task methods

The single-task methods considered in this study are: random forest (RF) and graph neural networks (GNN). RF uses a 1544-dimensional fingerprint-based reaction representation (Figure 4a). RF is implemented using the *scikit-learn* package. The hyperparameter tuning is done using the validation tasks. The parameters which did not result in a significant change in performance were set to their default values. The '*n_estimators*' hyperparameter is tuned and set to a value of 200.

The 1544-dimensional graph-based reaction representation are used with GNN (Figure 4b). This is passed through two fully-connected layers with dimensions 1024 and 512, followed by an output layer. A dropout rate of 0.1 is applied to these layers with PReLU activation function. The model is then trained for 400 epochs using the Adam optimizer with a learning rate of 0.001 and a batch size of 512. It is implemented using PyTorch.

1.2 Meta-learning methods

Prototypical network (ProtoNet) is the meta-learning method considered in this study. The model is implemented in PyTorch. We have used both the fingerprint and graph-based reaction representations as input features (Figures 4a and 4b). The 1544-dimensional input representation is passed through a neural network feature extractor with two-fully connected layers of dimensions 1024 and 512. A dropout rate of 0.1 is applied to these layers with PReLU activation function. The hyperparameters are optimized using the validation tasks. During meta-training, from each task we sample 512 reactions for the support set and 64 for query set. A total of five train tasks are used to get the average validation loss for optimization. The model is trained for 1000 epochs and validated after every 50 epochs. The model that provides the

best performance on the validation set is saved along with the fully trained model for making predictions on the test set. We use the Adam optimizer with a learning rate of 0.0001.

During the meta-training phase, the training tasks are divided into support and query sets. We use a support set size of 512, whereas query set size is 64. The model is first trained on the support set. The model parameters are then learned to obtain the best possible average validation loss (on the query set) over multiple training tasks (Figure 5b). In a single run, the validation loss is averaged over 5 training tasks. This results into a total of 2880 {(512+64)*5} reactions of $\mathcal{D}_{train}$ being utilized during one run. Since this process is repeated for multiple epochs, random subsets of $\mathcal{D}_{train}$ with 2880 reactions will be used. Thus, the full training process utilizes all the 9032 reactions of $\mathcal{D}_{train}$.

The 2400 reactions of $\mathcal{D}_{test}$ are divided randomly into 10 tasks, each comprising of 240 reactions. During meta-testing, these test tasks are partitioned into support and query sets. We have used three different support set sizes 16, 32, and 64. In each case, the query set size is fixed to 128. This results into a total of 144, 160, and 192 reactions being used when a test task is sampled to measure the model performance. We have used 10 different support and query random splits of every test task to report the average performance.

2. Model performance with fingerprint-based input representation

In this section, we compare the model performance of different meta-learning and single-task methods using the fingerprint-based reaction representation (Figure 4a). The performance is reported in terms of area under the precision-recall curve (AUPRC) as an average of ten random support-query splits. Additionally, the area under the receiver operating characteristic curve (AUROC) for all methods is also provided.

2.1 Performance of meta-learning method

The performance of prototypical networks in terms of AUPRC score is shown in Table S1.

Table S1. Model Performance of Prototypical Networks in Terms of AUROC and AUPRC Scores

Train-test split 1		
Support set size	AUROC	AUPRC
16	0.7523±0.0078	0.8092±0.0102
32	0.7757±0.0114	0.8442±0.0081
64	0.8120±0.0028	0.8661±0.0043
full training data	0.8796±0.0032	0.9133±0.0031
Train-test split 2		
Support set size	AUROC	AUPRC
16	0.7539±0.0427	0.8167±0.0465
32	0.7869±0.0120	0.8574±0.0069
64	0.8215±0.0094	0.8784±0.0144
full training data	0.8939±0.0046	0.9265±0.0051
Train-test split 3		
Support set size	AUROC	AUPRC
16	0.7613±0.0233	0.8096±0.0191
32	0.7843±0.0214	0.8590±0.0035
64	0.8319±0.0061	0.8685±0.0110
full training data	0.9017±0.0075	0.9171±0.0055
Train-test split 4		
Support set size	AUROC	AUPRC
16	0.7437±0.0314	0.8168±0.0290
32	0.8059±0.0098	0.8694±0.0136
64	0.8233±0.0110	0.8910±0.0133
full training data	0.8832±0.0057	0.9247±0.0075
Train-test split 5		
Support set size	AUROC	AUPRC
16	0.7552±0.0199	0.8242±0.0250
32	0.8124±0.0103	0.8696±0.0113
64	0.8286±0.0067	0.8760±0.0063
full training data	0.8781±0.0078	0.9209±0.0080

2.2 Performance of single-task methods

The performance of single-task methods in terms of AUPRC and AUROC score is shown in Table S2.

Table S2. Model Performance of RF in Terms of AUPRC and AUROC Scores

Train-test split 1

Support set size	AUROC	AUPRC
16	0.5049±0.0027	0.6437±0.0011
32	0.5182±0.0052	0.6534±0.0030
64	0.5352±0.0048	0.6625±0.0077
full training data	0.8120±0.0050	0.8369±0.0055
Train-test split 2		
Support set size	AUROC	AUPRC
16	0.5072±0.0046	0.6376±0.0122
32	0.5169±0.0071	0.6508±0.0070
64	0.5235±0.0074	0.6645±0.0100
full training data	0.8076±0.0086	0.8312±0.0094
Train-test split 3		
Support set size	AUROC	AUPRC
16	0.5023±0.0105	0.6362±0.0144
32	0.5232±0.0042	0.6429±0.0057
64	0.5451±0.0044	0.6578±0.0154
full training data	0.8003±0.0128	0.8146±0.0123
Train-test split 4		
Support set size	AUROC	AUPRC
16	0.5011±0.0037	0.6562±0.0097
32	0.5129±0.0080	0.6622±0.0052
64	0.5203±0.0030	0.6865±0.0126
full training data	0.8163±0.0085	0.8454±0.0083
Train-test split 5		
Support set size	AUROC	AUPRC
16	0.4944±0.0046	0.6326±0.0125
32	0.5302±0.0069	0.6395±0.0104
64	0.5194±0.0074	0.6526±0.0115
full training data	0.8179±0.0151	0.8445±0.0130

3. Model performance with graph-based input representation

In this section, we compare the model performance of different meta-learning and single-task methods using the graph-based reaction representation (Figure 4b). The performance is reported in terms of area under the precision-recall curve (AUPRC) as an average of ten random support-query splits. Additionally, the area under the receiver operating characteristic curve (AUROC) for all methods is also provided.

3.1 Performance of meta-learning method

The performance of prototypical networks in terms of AUPRC and AUROC scores is shown in Table S3.

Table S3. Model Performance of Prototypical Networks in Terms of AUPRC and AUROC Scores

Support set size	AUROC	AUPRC
16	0.7460±0.0039	0.8266±0.0032
32	0.7819±0.0007	0.8492±0.0005
64	0.8100±0.0009	0.8686±0.0011

3.2 Performance of single-task method

The performance of GNNs in terms of AUPRC and AUROC score is shown in Table S4.

Table S4. Model Performance of GNNs in Terms of AUPRC and AUROC Scores

Train-test split 1		
Support set size	AUROC	AUPRC
16	0.5278±0.0045	0.6550±0.0039
32	0.5244±0.0047	0.6577±0.0031
64	0.5535±0.0079	0.6622±0.0082
full training data	0.8021±0.0026	0.8259±0.0021
Train-test split 2		
Support set size	AUROC	AUPRC
16	0.5014±0.0108	0.6533±0.0130
32	0.5138±0.0087	0.6797±0.0218
64	0.5538±0.0057	0.6697±0.0121
full training data	0.8075±0.0124	0.8292±0.0146
Train-test split 3		
Support set size	AUROC	AUPRC
16	0.5324±0.0156	0.6836±0.0175
32	0.5359±0.0069	0.6862±0.0102
64	0.5492±0.0140	0.6853±0.0180
full training data	0.8054±0.0074	0.8291±0.0033
Train-test split 4		
Support set size	AUROC	AUPRC
16	0.5168±0.0082	0.6663±0.0101
32	0.5275±0.0267	0.6843±0.0159
64	0.5570±0.0058	0.6997±0.0094
full training data	0.8026±0.0063	0.8278±0.0052
Train-test split 5		

Support set size	AUROC	AUPRC
16	0.5224±0.0137	0.6455±0.0077
32	0.5310±0.0079	0.6613±0.0174
64	0.5440±0.0104	0.6487±0.0069
full training data	0.8073±0.0070	0.8197±0.0068

4. Dataset preparation for meta-cluster approach

In this section, we describe the process followed for constructing the tasks for meta-cluster approach. The full data consists of 11,932 AHO reactions. Out of these, the training set is comprised of 9032 reactions, while there are 2400 reactions in the test set. A total of 500 reactions are kept for the validation set. The dimensionality of the reaction feature space is reduced to 10 dimensions using the UMAP approach. This data is then used as an input to the clustering analysis. We use k-means clustering to identify the clusters present in the data. The elbow method is used to choose the optimal number of clusters with the elbow plot shown in Fig. S1. Although the Within-Cluster-Sum of Squared Error (WSS) decreases with the number of clusters, no sharp elbow is observed. Therefore, we choose k=15 as the optimal number of clusters as there is not much change in WSS with more clusters.

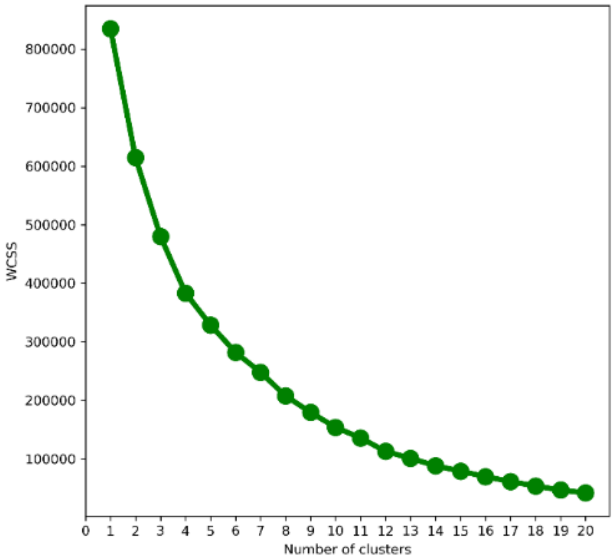

Fig. S1. Elbow plot to determine the optimal number of clusters.

In addition, we also performed the silhouette analysis which determines the separation distance between multiple clusters. The average silhouette score for different clusters is shown

in Table S5. A silhouette score closer to +1 is preferred as it indicates that samples are well separated from the neighboring clusters. With a maximum silhouette score of 0.68, k=4 is found to be the optimal number of clusters, which is similar to that obtained from the elbow plot.

Table S5. Silhouette Scores for Different Number of Clusters

Number of clusters	Average silhouette score
5	0.46
10	0.59
15	0.68
20	0.65

The number of reactions in each of the 15 clusters is shown in Table S6. It is noted that some of the clusters have lesser number of reactions. We merged these clusters to get a reasonable number of reactions in each cluster. This results into a total of ten clusters. Thus, there are 10 train tasks and 10 test tasks (Table S6).

Table S6. Distribution of Data into Various Clusters

<table>
<tr><td>C1</td><td>C2</td><td>C3</td><td>C4</td><td>C5</td><td>C6</td><td>C7</td><td>C8</td><td>C9</td><td>C10</td><td>C11</td><td>C12</td><td>C13</td><td>C14</td><td>C15</td></tr>
<tr><td colspan="15">training set</td></tr>
<tr><td>783</td><td>1224</td><td>263</td><td>228</td><td>568</td><td>346</td><td>482</td><td>706</td><td>2311</td><td>488</td><td>490</td><td>255</td><td>261</td><td>445</td><td>182</td></tr>
<tr><td colspan="15">test set</td></tr>
<tr><td>218</td><td>359</td><td>63</td><td>55</td><td>155</td><td>86</td><td>126</td><td>208</td><td>583</td><td>119</td><td>150</td><td>61</td><td>58</td><td>109</td><td>50</td></tr>
<tr><td colspan="15">After merging the clusters</td></tr>
<tr><td colspan="15">training set</td></tr>
<tr><td>C1</td><td>C2</td><td>C5</td><td>C8</td><td>C9</td><td colspan="2">C3+C7</td><td colspan="2">C4+C6</td><td colspan="2">C11+C12</td><td colspan="2">C11+C13</td><td colspan="2">C14+C15</td></tr>
<tr><td>783</td><td>1224</td><td>568</td><td>706</td><td>2311</td><td colspan="2">745</td><td colspan="2">574</td><td colspan="2">745</td><td colspan="2">751</td><td colspan="2">627</td></tr>
<tr><td colspan="15">test set</td></tr>
<tr><td>218</td><td>359</td><td>189</td><td>208</td><td>583</td><td colspan="2">189</td><td colspan="2">141</td><td colspan="2">180</td><td colspan="2">208</td><td colspan="2">159</td></tr>
</table>

The identity of reactions in each of the 10 clusters in terms of metal catalyst, type of olefins, and ligands is also studied. Almost every cluster has reactions comprising of all three metal catalysts, although in varying numbers. There is no specific pattern observed in terms of type of olefin or ligand among various clusters. The type of olefins and ligands majorly present in different clusters is shown in Table S7.

Table S7. Identity of Reactions in Terms of Metal Catalysts, Type of Olefin, and Ligand Present in Different Clusters

cluster	metal catalyst	olefin type	ligand type
C1	Ir, Rh, Co	aryl- and alkyl-substituted alkenes, enamides, α,β-unsaturated carbonyls	P,N ligands, P,P ligands, NNN-pincer
C2	Ir, Rh, Co	aryl- and alkyl-substituted alkenes, enamides, α,β-unsaturated carbonyls	P,N ligands, P,P ligands, mono P-ligands
C3	Ir	aryl- and alkyl-substituted alkenes	P,N ligands, P,O ligands
C4	Ir	aryl- and alkyl-substituted alkenes, α,β-unsaturated carbonyls, alkenes bearing heteroatoms	P,N ligands
C5	Rh	enamides, α,β-unsaturated carbonyls	P,N ligands, P,P ligands
C6	Ir, Rh, Co	aryl- and alkyl-substituted alkenes, α,β-unsaturated carbonyls, alkenes bearing heteroatoms	P,N ligands, P,P ligands, NNN-pincer, mono P-ligands, P,S ligands
C7	Ir, Rh	aryl- and alkyl-substituted alkenes, enamides, α,β-unsaturated carbonyls	P,N ligands, P,P ligands, mono P-ligands, P,S ligands
C8	Ir, Rh, Co	aryl- and alkyl-substituted alkenes, enamines and enamides, α,β-unsaturated carbonyls	P,N ligands, P,P ligands, mono P-ligands
C9	Ir, Rh	aryl- and alkyl-substituted alkenes, α,β-unsaturated carbonyls, alkenes bearing heteroatoms, enols	P,N ligands, P,P ligands, mono P-ligands
C10	Ir, Rh	aryl- and alkyl-substituted alkenes, enamides, α,β-unsaturated carbonyls	P,N ligands, P,P ligands, mono P-ligands

5. Model performance with meta-cluster approach

In this section, we compare the model performance of different meta-learning and single-task methods using the meta-cluster approach at meta-test time (Figure 7a). The performance is reported in terms of area under the precision-recall curve (AUPRC) as an average of ten random support-query splits. Additionally, the area under the receiver operating characteristic curve (AUROC) for all methods is also provided.

5.1 Performance of meta-learning method

The performance of prototypical networks in terms of AUPRC score is shown in Table S8.

Table S8. Model Performance of Prototypical Networks in Terms of AUROC and AUPRC Scores

Train-test split 1		
Support set size	AUROC	AUPRC
16	0.8032±0.0042	0.8602±0.0050
32	0.8345±0.0028	0.8901±0.0027
64	0.8630±0.0056	0.9108±0.0046
full cluster data	0.8934±0.0015	0.9300±0.0011
Train-test split 2		
Support set size	AUROC	AUPRC
16	0.7923±0.0046	0.8567±0.0048
32	0.8367±0.0027	0.8920±0.0026
64	0.8625±0.0059	0.9126±0.0061
full cluster data	0.8924±0.0005	0.9312±0.0010
Train-test split 3		
Support set size	AUROC	AUPRC
16	0.7880±0.0078	0.8385±0.0101
32	0.8330±0.0030	0.8850±0.0029
64	0.8573±0.0020	0.9051±0.0032
full cluster data	0.8940±0.0024	0.9276±0.0022
Train-test split 4		
Support set size	AUROC	AUPRC
16	0.8117±0.0099	0.8827±0.0067
32	0.8287±0.0042	0.8961±0.0027
64	0.8605±0.0038	0.9147±0.0023
full cluster data	0.8918±0.0021	0.9291±0.0015
Train-test split 5		
Support set size	AUROC	AUPRC
16	0.7806±0.0056	0.8466±0.0037
32	0.8265±0.0024	0.8778±0.0028
64	0.8583±0.0022	0.9007±0.0014
full cluster data	0.8922±0.0017	0.9314±0.0005

For an additional insight into the model performance, the confusion matrices for all support set sizes are also reported in Table S9.

Table S9. Model Performance in Terms of Confusion Matrices for Prototypical Networks

Support set size		
16	2198	1438
	980	5624

32	2398	1282
	958	5602
64	2533	1189
	983	5535
full cluster data	2727	965
	851	5697
full training data	2633	1110
	796	5701

The performance of prototypical networks without neural network embedding function is provided in Table S10. It can be noted from Table S10 that the performance of prototypical networks without the embedding function is considerably lower as compared to with neural network (Table S8).

Table S10. Model Performance of Prototypical Networks Without Neural Network in Terms of AUROC and AUPRC Scores

Support set size	AUROC	AUPRC
16	0.6092±0.0099	0.7219±0.0061
32	0.6342±0.0075	0.7416±0.0079
64	0.6894±0.0037	0.7680±0.0043
full cluster data	0.8205±0.0018	0.8589±0.0019

5.2 Performance of single-task methods

The performance of single-task methods in terms of AUPRC and AUROC score is shown in Table S11. The performance of gradient boosting (XGBoost) and support vector classification (SVC) is found to be lower when compared to RF and GNN.

Table S11. Model Performance of Single-task Methods in Terms of AUPRC and AUROC Scores

	RF	
Support set size	AUROC	AUPRC
16	0.5534±0.0052	0.6742±0.0046

32	0.5865±0.0033	0.6875±0.0034
64	0.6324±0.0067	0.7150±0.0037
full cluster data	0.8068±0.0038	0.8281±0.0048
	GNN	
Support set size	AUROC	AUPRC
16	0.5596±0.0045	0.6745±0.0068
32	0.5886±0.0033	0.6829±0.0023
64	0.6360±0.0061	0.7144±0.0043
full cluster data	0.7906±0.0044	0.8199±0.0047
	XGBoost	
Support set size	AUROC	AUPRC
16	0.5404±0.0015	0.6602±0.0014
32	0.5812±0.0031	0.6801±0.0022
64	0.6187±0.0009	0.7025±0.0011
full cluster data	0.7798±0.0028	0.8087±0.0040
full training data	0.6739±0.0042	0.7311±0.0022
	SVC	
Support set size	AUROC	AUPRC
16	0.5407±0.0074	0.6636±0.0028
32	0.5533±0.0050	0.6667±0.0026
64	0.5703±0.0105	0.6711±0.0050
full cluster data	0.7775±0.0017	0.8005±0.0030
full training data	0.7688±0.0027	0.7981±0.0020

The performance of RF and GNN on five different train-test splits is provided in Table S12.

Table S12. Model Performance of RF and GNN in Terms of AUPRC and AUROC Scores on Five Train-Test Splits

RF		
	Train-test split 1	
Support set size	AUROC	AUPRC
16	0.5534±0.0052	0.6742±0.0046
32	0.5865±0.0033	0.6875±0.0034
64	0.6324±0.0067	0.7150±0.0037
full cluster data	0.8068±0.0038	0.8281±0.0048
	Train-test split 2	
Support set size	AUROC	AUPRC
16	0.5604±0.0094	0.6825±0.0055
32	0.5876±0.0059	0.6940±0.0028
64	0.6326±0.0076	0.7152±0.0042
full cluster data	0.7960±0.0046	0.8255±0.0051

	Train-test split 3	
Support set size	AUROC	AUPRC
16	0.5558±0.0077	0.6668±0.0045
32	0.6006±0.0024	0.6824±0.0030
64	0.6301±0.0062	0.7005±0.0037
full cluster data	0.7914±0.0027	0.8113±0.0023
	Train-test split 4	
Support set size	AUROC	AUPRC
16	0.5534±0.0027	0.6963 +- 0.0021
32	0.5822±0.0029	0.7073 +- 0.0015
64	0.6220±0.0020	0.7326 +- 0.0026
full cluster data	0.8040± 0.0023	0.8461 +- 0.0027
	Train-test split 5	
Support set size	AUROC	AUPRC
16	0.5603±0.0034	0.6768±0.0053
32	0.5795±0.0092	0.6832±0.0041
64	0.6332±0.0091	0.7152±0.0057
full cluster data	0.7927±0.0009	0.8145±0.0039
GNN		
	Train-test split 1	
Support set size	AUROC	AUPRC
16	0.5596±0.0045	0.6745±0.0068
32	0.5886±0.0033	0.6829±0.0023
64	0.6360±0.0061	0.7144±0.0043
full cluster data	0.7906±0.0044	0.8199±0.0047
	Train-test split 2	
Support set size	AUROC	AUPRC
16	0.5552±0.0039	0.6837±0.0052
32	0.5831±0.0078	0.6865±0.0059
64	0.6301±0.0033	0.7224±0.0047
full cluster data	0.7917±0.0046	0.8265±0.0046
	Train-test split 3	
Support set size	AUROC	AUPRC
16	0.5700±0.0061	0.6982±0.0079
32	0.5823±0.0030	0.6971±0.0023
64	0.6172±0.0068	0.7202±0.0060
full cluster data	0.7924±0.0014	0.8339±0.0022
	Train-test split 4	
Support set size	AUROC	AUPRC
16	0.5642±0.0037	0.6878±0.0063
32	0.5818±0.0083	0.7053±0.0040
64	0.6360±0.0038	0.7302±0.0029

full cluster data	0.7878±0.0038	0.8281±0.0032
	Train-test split 5	
Support set size	AUROC	AUPRC
16	0.5438±0.0096	0.6828±0.0047
32	0.5850±0.0036	0.7029±0.0028
64	0.6196±0.0043	0.7230±0.0033
full cluster data	0.7795±0.0021	0.8233±0.0028

6. Model performance with different classification thresholds

We have also evaluated the performance of meta-cluster approach with different classification threshold. Two classification thresholds are used: 70 and 90. The result of Prototypical networks and RF in terms of AUPRC and AUROC score is shown in Table S13.

Table S13. Model Performance of Prototypical Networks and RF with Different Classification Thresholds

Classification threshold = 70		
	Prototypical networks	
Support set size	AUROC	AUPRC
16	0.7551±0.0093	0.8628±0.0058
32	0.8199±0.0025	0.9042±0.0031
64	0.8397±0.0047	0.9199±0.0020
full cluster data	0.8653±0.0055	0.9302±0.0049
full training data	0.8436±0.0035	0.9166±0.0024
	RF	
Support set size	AUROC	AUPRC
16	0.5400±0.0046	0.7421±0.0052
32	0.5505±0.0025	0.7401±0.0024
64	0.5852±0.0041	0.7577±0.0023
full cluster data	0.7635±0.0043	0.8450±0.0032
full training data	0.7373±0.0027	0.8300±0.0012
Classification threshold = 90		
	Prototypical networks	
Support set size	AUROC	AUPRC
16	0.8190±0.0091	0.8028±0.0077
32	0.8454±0.0026	0.8259±0.0026
64	0.8642±0.0029	0.8474±0.0030
full cluster data	0.8959±0.0022	0.8765±0.0030
full training data	0.8906±0.0074	0.8731±0.0114
	RF	

Support set size	AUROC	AUPRC
16	0.5662±0.0096	0.5358±0.0073
32	0.6205±0.0040	0.5771±0.0026
64	0.6673±0.0050	0.6091±0.0053
full cluster data	0.8288±0.0039	0.7647±0.0051
full training data	0.8389±0.0026	0.7734±0.0031

7. Identity of predictions

We have also analyzed the predictions in terms of metal catalyst, type of olefin and ligand. For this purpose, we have chosen the Protonet_cluster model. The predictions of one of the test runs is considered. The details are provided in Table S14. No particular trend is observed in type of reactions that are challenging to make predictions for.

Table S14. Identity of Predictions in Terms of Metal Catalyst, Type of Olefin and Ligand

y_true	y_pred	metal	olefin_type		ligand_type
0	0	Rh	tri-sub	α,β-unsaturated ketone	bisphosphine
0	0	Ir	tetra-sub	α,β-unsaturated acid	phosphine-oxazoline
0	0	Rh	tri-sub	α,β-unsaturated ester	bisphosphine
0	0	Rh	tri-sub	enamide	phosphoramidite
0	0	Rh	tri-sub	enamide	bisphosphine
0	0	Rh	tri-sub	α,β-unsaturated amide	bisphosphine
0	0	Ir	tetra-sub	α,β-unsaturated ester	N-phosphine-oxazoline
0	0	Rh	tri-sub	phosphorous	bisphosphine
0	0	Rh	tri-sub	α,β-unsaturated ester	bisphosphine
0	0	Rh	di-sub	enamide	phosphine
0	0	Rh	tetra-sub	enamide	phosphine
0	0	Ir	tetra-sub	aryl-alkyl	N-phosphine-oxazoline
0	0	Rh	tri-sub	α,β-unsaturated ester	ferrocene bisphosphine
0	0	Ir	tetra-sub	α,β-unsaturated ester	phosphine-imidazole
0	0	Rh	tri-sub	aryl-alkyl-NO2	ferrocene bisphosphine
0	0	Rh	di-sub	enamide	biaryl phosphine
0	0	Rh	tri-sub	α,β-unsaturated ester	bisphosphine
0	0	Rh	tri-sub	α,β-unsaturated ester	ferrocene bisphosphine

0	0	Rh	tri-sub	α,β-unsaturated ketone	bisphosphine
0	0	Ir	tri-sub	α,β-unsaturated ketone	phosphine-N-donor
0	0	Rh	tri-sub	α,β-unsaturated amide	ferrocene bisphosphine
0	0	Rh	di-sub	enamide	biaryl phosphine
0	0	Ir	tri-sub	allyl alcohol	phosphine-N-donor
0	0	Ir	tetra-sub	α,β-unsaturated ester	bisphosphine
0	0	Rh	tri-sub	enamide	ferrocene bisphosphine
0	0	Ir	tetra-sub	α,β-unsaturated ester	N-phosphine-thiazole
0	0	Ir	di-sub	α,β-unsaturated acid	phosphine-oxazoline
0	0	Rh	di-sub	aryl-alkyl (Py)	bisphosphine
0	0	Rh	tri-sub	enamide	phosphoramidite-phosphoramidite
0	0	Rh	tri-sub	enamide	bisphosphine
0	0	Rh	tri-sub	enamide	bisphosphine
0	0	Rh	tri-sub	enamide	phosphoramidite-phosphoramidite
0	0	Rh	tri-sub	enamide	bisphosphine
0	0	Rh	di-sub	α,β-unsaturated acid	ferrocene bisphosphine
0	0	Rh	di-sub	enamide	bisphosphine
0	0	Ir	tri-sub	sulphur(SO2)	phosphinite-pyridine
0	0	Rh	tri-sub	α,β-unsaturated acid	bisphosphine
0	0	Rh	tri-sub	enamide	phosphoramidite
0	0	Rh	tri-sub	enamide	ferrocene bisphosphine
0	0	Ir	di-sub	aryl-alkyl	phosphinite-oxazoline
0	0	Rh	di-sub	enamide	phosphonite
0	0	Rh	di-sub	enamide	phosphoramidite
0	0	Rh	di-sub	enamide	phosphoramidite
0	0	Rh	tri-sub	enamide	phoshite
0	0	Rh	tri-sub	enamide	phosphoramidite- phosphite
0	0	Rh	tri-sub	aryl-alkyl	ferrocene bisphosphine
0	0	Rh	tri-sub	enamide	phosphoramidite-phosphoramidite
0	0	Ir	di-sub	aryl-alkyl	phosphite-pyridine
0	0	Ir	tri-sub	α,β-unsaturated amide	phosphine-oxazoline
0	0	Rh	di-sub	enamide	phosphoramidite
0	0	Rh	di-sub	α,β-unsaturated acid	phosphoramidite
0	0	Rh	tri-sub	enamide	phosphoramidite

0	0	Ir	tri-sub	α,β-unsaturated ester	phosphine-oxazoline
0	0	Ir	tri-sub	aryl-alkyl	phosphine-carbene
0	0	Ir	tri-sub	aryl-alkyl	ferrocene mono phosphine
0	0	Ir	tri-sub	aryl-alkyl	phosphite-pyridine
0	0	Ir	tri-sub	allyl ester	phosphine-pyridine
0	0	Ir	tri-sub	allyl ester	ferrocene-phosphine-N-donor
0	0	Ir	tri-sub	enol ether	phosphine-imidazole
0	0	Ir	tri-sub	allyl alcohol	phosphine-carbene
0	0	Ir	tri-sub	aryl-alkyl-N	phosphine-N-donor
0	0	Ir	tri-sub	aryl-alkyl	phosphine-imidazole
0	0	Ir	tetra-sub	aryl-alkyl	phosphite-oxazoline
0	0	Ir	tri-sub	allyl alcohol	phosphinite-oxazoline
0	0	Ir	tri-sub	aryl-alkyl	phosphite-oxazoline
0	0	Ir	tri-sub	aryl-alkyl	phosphinite-oxazoline
0	0	Ir	tri-sub	aryl-alkyl	phosphite-pyridine
0	0	Ir	tri-sub	α,β-unsaturated ketone	phosphine-oxazoline
0	0	Ir	tri-sub	α,β-unsaturated ester	phosphoramidite-pyridine
0	0	Ir	tri-sub	allyl alcohol	phosphine-carbene
0	0	Ir	di-sub	α,β-unsaturated ester	phosphine-carbene
0	0	Rh	tri-sub	α,β-unsaturated ester	bisphosphine
0	0	Ir	tri-sub	aryl-alkyl-N	phosphine-imidazole
0	0	Ir	tri-sub	aryl-alkyl-N	phosphine-pyridine
0	0	Ir	tri-sub	aryl-alkyl	phosphine-oxazoline
0	0	Ir	tri-sub	α,β-unsaturated ester	phosphinite-pyridine
0	0	Ir	tri-sub	enol ether	phosphine-imidazole
0	0	Ir	tri-sub	enol ether	phosphine-imidazole
0	0	Ir	tri-sub	aryl-alkyl-N	phosphine-carbene
0	0	Ir	tetra-sub	aryl-alkyl	phosphine-N-donor
0	0	Ir	tri-sub	enol ether	phosphine-imidazole
0	0	Ir	tri-sub	α,β-unsaturated ester	phosphinite-oxazoline
0	0	Ir	tri-sub	aryl-alkyl	phosphine-oxazoline
0	0	Ir	tri-sub	allyl ester	phosphine-pyridine
0	0	Ir	tetra-sub	aryl-alkyl	phosphite-oxazoline
0	0	Ir	tri-sub	α,β-unsaturated ester	phosphinite-pyridine
0	0	Ir	tri-sub	enol ether	phosphine-imidazole
0	0	Ir	di-sub	aryl-alkyl	phosphite-oxazoline
0	0	Ir	tri-sub	aryl-alkyl	phosphine-imidazole
0	0	Ir	tri-sub	α,β-unsaturated ester	phosphine-pyridine

0	0	Ir	tri-sub	sulphur	phosphine-oxazoline
0	0	Ir	tri-sub	aryl-alkyl-N	phosphine-imidazole
0	0	Ir	tri-sub	aryl-alkyl	N-phosphine-oxazoline
0	0	Ir	di-sub	aryl-alkyl	phosphinite-oxazoline
0	0	Ir	tri-sub	allyl alcohol	phosphinite-pyridine
0	0	Ir	tri-sub	aryl-alkyl	N-phosphine-oxazoline
0	0	Ir	tri-sub	aryl-alkyl	phosphite-pyridine
0	0	Ir	tri-sub	aryl-alkyl	phosphine-imidazole
0	0	Ir	di-sub	aryl-alkyl	phosphite-oxazoline
0	0	Ir	tri-sub	allyl alcohol	phosphine-O
0	0	Rh	tri-sub	enamide	biaryl bisphosphine
0	0	Ir	tri-sub	boron	phosphine-pyridine
0	0	Ir	tri-sub	α,β-unsaturated ester	ferrocene-phosphine-N-donor
0	0	Ir	tri-sub	α,β-unsaturated ester	phosphine-pyridine
0	0	Ir	tri-sub	allyl ester	phosphine-pyridine
0	0	Ir	tri-sub	α,β-unsaturated ester	phosphine-thiazole
0	0	Ir	di-sub	allyl ester	phosphite-pyridine
0	0	Rh	di-sub	α,β-unsaturated ester	phosphite
0	0	Ir	di-sub	enamide	phosphine-N-donor
0	0	Rh	di-sub	enol ester	phosphite
0	0	Ir	tri-sub	boron	phoshine-oxazoline
0	0	Ir	tri-sub	aryl-alkyl	N-phosphine-thiazole
0	0	Ir	tri-sub	enamine	phosphoramidite
0	0	Rh	di-sub	α,β-unsaturated ester	phosphoramidite-phosphoramidite
0	0	Rh	di-sub	enamide	phosphoramidite-phosphoramidite
0	0	Ir	di-sub	aryl-alkyl	N-phosphine-thiazole
0	0	Ir	tri-sub	aryl-alkyl-N	phosphine-oxazoline
0	0	Ir	tri-sub	allyl ester	phosphine-pyridine
0	0	Ir	tri-sub	boron	N-phosphine-imidazole
0	0	Ir	di-sub	aryl-alkyl	N-phosphine-oxazoline
0	0	Ir	tri-sub	aryl-alkyl-N	phosphine-oxazoline
0	0	Rh	di-sub	enol ester	phosphite
0	0	Ir	di-sub	silicon	phosphinite-pyridine
0	0	Ir	tri-sub	silicon	phosphinite-imidazole
0	0	Ir	tri-sub	boron	phosphine-oxazoline
0	0	Ir	tri-sub	allyl alcohol	phosphine-pyridine
0	0	Rh	tetra-sub	enamide	phosphoramidite
0	0	Rh	di-sub	α,β-unsaturated ester	phosphoramidite
0	0	Rh	tri-sub	aryl-alkyl-NO2	bisphosphine
0	0	Ir	tri-sub	α,β-unsaturated ester	phosphine-N-donor

0	0	Ir	tri-sub	aryl-alkyl-N	phosphine-carbene
0	0	Ir	tri-sub	α,β-unsaturated ester	phosphite-oxazoline
0	0	Rh	tri-sub	aryl-alkyl-NO2	ferrocene bisphosphine
0	0	Rh	tri-sub	enamide	biaryl phosphine-biaryl phosphine-ferrocene
0	0	Rh	di-sub	boron	bisphosphine
0	0	Ir	tri-sub	aryl-alkyl-N	phosphine-N-donor
0	0	Rh	di-sub	enamide	phosphoramidite-pyridine
0	0	Rh	tri-sub	enamide	phosphoramidite
0	0	Ir	tetra-sub	enamide	ferrocene bisphosphine
0	0	Rh	tri-sub	α,β-unsaturated amide	bisphosphine
0	0	Rh	tri-sub	enamide	ferrocene bisphosphine
0	0	Rh	tri-sub	aryl-alkyl-NO2	ferrocene bisphosphine
0	0	Ir	tri-sub	aryl-alkyl	phosphite-thioether
0	0	Ir	tri-sub	aryl-alkyl	phosphite-thioether
0	0	Rh	tri-sub	α,β-unsaturated ketone	bisphosphine
0	0	Rh	tri-sub	α,β-unsaturated ester	ferrocene bisphosphine
0	0	Co	di-sub	aryl-alkyl-N	bisphosphine
0	0	Rh	tri-sub	enamide	phosphoramidite-phosphoramidite
0	0	Rh	tri-sub	enamide	bisphosphine
0	0	Rh	tri-sub	enamide	phosphoramidite
0	0	Co	tetra-sub	α,β-unsaturated acid	bisphosphine
0	0	Rh	tri-sub	α,β-unsaturated ester	ferrocene bisphosphine
0	0	Ir	tri-sub	α,β-unsaturated ester	phosphine-N-donor
0	0	Ir	tri-sub	aryl-alkyl	phosphine-thioether
0	0	Ir	tri-sub	aryl-alkyl	phosphite-thioether
0	0	Rh	tri-sub	aryl-alkyl-NO2	ferrocene bisphosphine
0	0	Rh	di-sub	α,β-unsaturated ester	phosphoramidite
0	0	Ir	tri-sub	aryl-alkyl	phosphite-thioether
0	0	Ir	tri-sub	aryl-alkyl	phosphinite-thioether
0	0	Ir	tri-sub	aryl-alkyl	phosphite-thioether
0	0	Rh	tri-sub	enamide	phosphoramidite-phosphoramidite
0	0	Ir	tri-sub	α,β-unsaturated ketone	phosphite-oxazoline
0	0	Rh	tri-sub	α,β-unsaturated ester	ferrocene bisphosphine
0	0	Rh	tri-sub	α,β-unsaturated ketone	bisphosphine

0	0	Ir	tri-sub	aryl-alkyl	phosphite-thioether
0	0	Ir	tri-sub	aryl-alkyl	phosphite-thioether
0	0	Co	tri-sub	enamide	bisphosphine
0	0	Co	tri-sub	enamide	bisphosphine
0	0	Rh	tri-sub	enamide	phosphine
0	0	Rh	tri-sub	enamide	bisphosphine
0	0	Ir	di-sub	aryl-alkyl	phosphinite-thioether
0	0	Ir	tri-sub	aryl-alkyl-N	phosphine-carbene
0	0	Ir	di-sub	aryl-alkyl	phosphite-thioether
0	0	Rh	tri-sub	enamide	phosphoramidite
0	0	Ir	di-sub	aryl-alkyl	phosphinite-thioether
0	0	Rh	tri-sub	enamide	phosphoramidite
0	0	Rh	tri-sub	enamide	bisphosphine
0	0	Ir	tetra-sub	aryl-alkyl	phosphine-oxazoline
0	0	Rh	tri-sub	enamide	phosphoramidite
0	0	Ir	di-sub	aryl-alkyl	phosphite-pyridine
0	0	Ir	tetra-sub	aryl-alkyl	phosphinite-oxazoline
0	0	Rh	tri-sub	enamide	bisphosphine
0	0	Rh	tri-sub	enamide	phosphonite
0	0	Rh	tri-sub	enamide	phosphoramidite
0	0	Ir	di-sub	aryl-alkyl	phosphine-thioether
0	0	Rh	di-sub	enamide	phosphoramidite
0	0	Rh	tri-sub	enamide	phosphine-carbene
0	0	Rh	tri-sub	enamide	phosphoramidite
0	0	Ir	tri-sub	aryl-alkyl	phosphite-oxazoline
0	0	Ir	tri-sub	aryl-alkyl	phosphine-oxazoline
0	0	Rh	tri-sub	enamide	bisphosphine
0	0	Rh	tetra-sub	enamide	phosphoramidite
0	0	Ir	tri-sub	aryl-alkyl	phosphite-oxazoline
0	0	Ir	tri-sub	allyl alcohol	ferrocene-phosphine-N-donor
0	0	Ir	di-sub	aryl-alkyl	phosphite-pyridine
0	0	Rh	tri-sub	enamide	bisphosphine
0	0	Ir	tetra-sub	aryl-alkyl	phosphinite-oxazoline
0	0	Ir	di-sub	aryl-alkyl	phosphite-thioether
0	0	Ir	tri-sub	allyl alcohol	ferrocene-phosphine-N-donor
0	0	Rh	di-sub	enamide	phosphoramidite
0	0	Rh	tri-sub	sulphur(SO2)	bisphosphine
0	0	Rh	tri-sub	enamide	phosphoramidite
0	0	Rh	tri-sub	enamide	phosphine-carbene
0	0	Ir	tri-sub	aryl-alkyl-N	phosphinite-pyridine
0	0	Rh	di-sub	α,β-unsaturated ester	phosphoramidite
0	0	Rh	tri-sub	sulphur(SO2)	bisphosphine
0	0	Ir	tri-sub	α,β-unsaturated amide	phosphine-oxazoline

0	0	Ir	di-sub	aryl-alkyl	monophosphinite
0	0	Ir	tri-sub	α,β-unsaturated amide	phosphine-oxazoline
0	0	Rh	tri-sub	enamide	phosphoramidite
0	0	Ir	di-sub	aryl-alkyl	monophosphinite-thiooether
0	0	Rh	tri-sub	enamide	bisphosphine
0	0	Ir	tetra-sub	aryl-alkyl	phosphine-oxazoline
0	0	Ir	di-sub	aryl-alkyl	phosphite-thioether
0	0	Rh	tetra-sub	enamide	phosphoramidite
0	0	Ir	di-sub	aryl-alkyl	phosphite-pyridine
0	0	Rh	tri-sub	enamide	phosphonite
0	0	Rh	tetra-sub	enamide	phosphoramidite
0	0	Rh	tri-sub	α,β-unsaturated ester	biaryl phosphine
0	0	Rh	di-sub	enol carbamate	biaryl phosphine
0	0	Rh	tetra-sub	enamide	bisphosphine
0	0	Rh	tri-sub	enamide	phosphite-phosphine
0	0	Rh	tri-sub	enamide	bisphosphine
0	0	Rh	tri-sub	enamide	phosphoramidite
0	0	Ir	di-sub	enamide	phosphoramidite
0	0	Ir	di-sub	enamine	phosphoramidite
0	0	Rh	tri-sub	enamide	biaryl phosphine
0	0	Rh	tri-sub	α,β-unsaturated ester	bisphosphine
0	0	Rh	tri-sub	α,β-unsaturated acid	monodentate phosphine oxide
0	0	Rh	di-sub	enol ester	phosphite-phosphine
0	0	Rh	tri-sub	enamide	bisphosphine
0	0	Rh	tri-sub	α,β-unsaturated ester	bisphosphine
0	0	Rh	tri-sub	enamide	biaryl phosphine
0	0	Rh	tri-sub	enamide	ferrocene bisphosphine
0	0	Rh	tri-sub	enamide	phosphite-phosphine
0	0	Rh	tri-sub	enamide	biaryl phosphine
0	0	Rh	tri-sub	α,β-unsaturated acid	monodentate phosphine oxide
0	0	Rh	tetra-sub	enamide	biaryl bisphosphine
0	0	Ir	tri-sub	aryl-alkyl-N	phosphine-carbene
0	0	Rh	di-sub	enamide	phosphoramidite
0	0	Ir	tri-sub	α,β-unsaturated ester	phosphine-oxazoline
0	0	Ir	di-sub	enamine	phosphoramidite
0	0	Rh	di-sub	enamide	phosphoramidite
0	0	Rh	tri-sub	enamide	biaryl phosphine
0	0	Rh	di-sub	enol carbamate	biaryl phosphine
0	0	Ir	tri-sub	α,β-unsaturated amide	bisphosphine
0	0	Rh	tri-sub	enamide	biaryl phosphine

0	0	Ir	tri-sub	α,β-unsaturated ketone	phosphine-oxazoline
0	0	Ir	tri-sub	α,β-unsaturated amide	phosphine-oxazoline
0	0	Rh	tri-sub	enamide	biaryl phosphine
0	0	Rh	tri-sub	enamide	biaryl phosphine
0	0	Rh	tetra-sub	enamide	phosphoramidite
0	0	Rh	tri-sub	enamide	biaryl phosphine
0	0	Rh	tri-sub	α,β-unsaturated ester	bisphosphine
0	0	Rh	di-sub	enol carbamate	biaryl phosphine
0	0	Rh	tri-sub	enamide	ferrocene bisphosphine
0	0	Rh	di-sub	α,β-unsaturated ester	bisphosphine
0	0	Rh	tri-sub	enamide	ferrocene bisphosphine
0	0	Rh	di-sub	α,β-unsaturated ester	phosphoramidite
0	0	Rh	di-sub	enol ester	phosphite
0	0	Rh	di-sub	α,β-unsaturated ester	phosphoramidite
0	0	Ir	di-sub	boron	N-phosphine-oxazoline
0	0	Ir	di-sub	aryl-alkyl	phosphinite-oxazoline
0	0	Ir	tri-sub	aryl-alkyl-N	phosphinite-pyridine
0	0	Ir	di-sub	aryl-alkyl	phosphite-oxazoline
0	0	Rh	di-sub	α,β-unsaturated ester	bisphosphine
0	0	Ir	di-sub	aryl-alkyl	phosphite-pyridine
0	0	Rh	tri-sub	enamide	bisphosphine
0	0	Rh	di-sub	enamide	phosphite
1	1	Co	di-sub	enamide	bisphosphine
1	1	Co	di-sub	enamide	bisphosphine
1	1	Co	tri-sub	aryl-alkyl	NNN-pincer
1	1	Co	di-sub	enamide	bisphosphine
1	1	Co	tri-sub	aryl-alkyl	NNN-pincer
1	1	Co	di-sub	enamide	bisphosphine
1	1	Co	tri-sub	enamide	bisphosphine
1	1	Co	di-sub	enamide	bisphosphine
1	1	Co	tetra-sub	α,β-unsaturated acid	bisphosphine
1	1	Co	tri-sub	enamide	bisphosphine
1	1	Co	di-sub	aryl-alkyl-N	bisphosphine
1	1	Co	tri-sub	α,β-unsaturated acid	bisphosphine
1	1	Co	tri-sub	aryl-alkyl	NNN-pincer
1	1	Co	di-sub	aryl-alkyl-N	bisphosphine
1	1	Co	di-sub	aryl-alkyl-N	bisphosphine
1	1	Co	tetra-sub	α,β-unsaturated acid	bisphosphine

1	1	Co	tetra-sub	α,β-unsaturated acid	bisphosphine
1	1	Co	tetra-sub	α,β-unsaturated acid	bisphosphine
1	1	Co	tetra-sub	α,β-unsaturated acid	bisphosphine
1	1	Co	di-sub	aryl-alkyl	NNN-pincer
1	1	Co	di-sub	silicon	phosphine-pyridine-oxazoline(PPO)
1	1	Co	di-sub	aryl-alkyl	NNN-pincer
1	1	Co	tri-sub	α,β-unsaturated acid	bisphosphine
1	1	Co	di-sub	silicon	phosphine-pyridine-oxazoline(PPO)
1	1	Co	tri-sub	aryl-alkyl	NNN-pincer
1	1	Co	di-sub	α,β-unsaturated acid	bisphosphine
1	1	Co	tri-sub	boron	NNN-pincer
1	1	Co	tri-sub	enamide	bisphosphine
1	1	Co	tri-sub	enamide	bisphosphine
1	1	Co	tri-sub	α,β-unsaturated acid	bisphosphine
1	1	Co	tri-sub	enamide	bisphosphine
1	1	Co	di-sub	enamide	bisphosphine
1	1	Co	tri-sub	α,β-unsaturated acid	bisphosphine
1	1	Co	tri-sub	α,β-unsaturated acid	bisphosphine
1	1	Co	tri-sub	α,β-unsaturated acid	bisphosphine
1	1	Co	di-sub	silicon	phosphine-pyridine-oxazoline(PPO)
1	1	Co	tri-sub	enamide	bisphosphine
1	1	Co	di-sub	α,β-unsaturated acid	bisphosphine
1	1	Co	tri-sub	aryl-alkyl	NNN-pincer
1	1	Co	di-sub	aryl-alkyl	NNN-pincer
1	1	Co	tri-sub	α,β-unsaturated acid	bisphosphine
1	1	Co	di-sub	silicon	phosphine-pyridine-oxazoline(PPO)
1	1	Co	tri-sub	enamide	bisphosphine
1	1	Co	tri-sub	α,β-unsaturated acid	bisphosphine
1	1	Co	tri-sub	α,β-unsaturated acid	bisphosphine
1	1	Ir	tetra-sub	α,β-unsaturated ester	N-phosphine-oxazoline

1	1	Ir	tetra-sub	α,β-unsaturated acid	phosphine-thiazole
1	1	Ir	tri-sub	α,β-unsaturated acid	phosphine-oxazoline
1	1	Ir	tri-sub	α,β-unsaturated acid	phosphine-oxazoline
1	1	Ir	tri-sub	α,β-unsaturated ketone	phosphine-N-donor
1	1	Ir	tri-sub	allyl alcohol	phosphine-N-donor
1	1	Ir	tetra-sub	α,β-unsaturated ester	N-phosphine-oxazoline
1	1	Ir	tri-sub	α,β-unsaturated acid	phosphine-oxazoline
1	1	Ir	di-sub	phosphorous	phosphine-oxazoline
1	1	Ir	tri-sub	α,β-unsaturated amide	phosphine-oxazoline
1	1	Ir	tetra-sub	α,β-unsaturated ester	N-phosphine-oxazoline
1	1	Ir	tri-sub	enol ether	phosphine-pyridine
1	1	Ir	tri-sub	enol ether	phosphine-pyridine
1	1	Ir	tri-sub	α,β-unsaturated acid	phosphine-oxazoline
1	1	Ir	tri-sub	aryl-alkyl	N-phosphine-thiazole
1	1	Ir	tri-sub	α,β-unsaturated ketone	phosphine-N-donor
1	1	Ir	tri-sub	enol ether	phosphine-pyridine
1	1	Ir	tri-sub	enol ether	phosphine-pyridine
1	1	Ir	tri-sub	α,β-unsaturated acid	phosphine-oxazoline
1	1	Ir	tetra-sub	α,β-unsaturated ester	ferrocene bisphosphine
1	1	Ir	tetra-sub	α,β-unsaturated ester	phosphine-thiazole
1	1	Ir	di-sub	α,β-unsaturated acid	phosphine-O
1	1	Ir	tetra-sub	α,β-unsaturated ester	bisphosphine
1	1	Ir	tri-sub	α,β-unsaturated amide	phosphine-oxazoline
1	1	Ir	tri-sub	α,β-unsaturated ketone	phosphine-oxazoline
1	1	Ir	di-sub	aryl-alkyl	phosphinite-oxazoline
1	1	Ir	tri-sub	α,β-unsaturated acid	phosphine-oxazoline
1	1	Ir	di-sub	aryl-alkyl	phosphinite-oxazoline
1	1	Ir	tri-sub	α,β-unsaturated ketone	phosphine-oxazoline
1	1	Ir	tri-sub	aryl-alkyl-N	phosphine-oxazoline

1	1	Ir	tri-sub	α,β-unsaturated amide	phosphine-oxazoline
1	1	Ir	tri-sub	α,β-unsaturated ketone	phosphine-oxazoline
1	1	Ir	di-sub	aryl-alkyl	phosphinite-oxazoline
1	1	Ir	tri-sub	α,β-unsaturated ester	phosphine-oxazoline
1	1	Ir	di-sub	aryl-alkyl	phosphite-oxazoline
1	1	Ir	di-sub	aryl-alkyl	phosphinite-oxazoline
1	1	Ir	tri-sub	α,β-unsaturated ester	phosphoramidite-pyridine
1	1	Ir	tri-sub	aryl-alkyl-N	phosphine-O
1	1	Ir	tri-sub	allyl alcohol	phosphinite-oxazoline
1	1	Ir	tri-sub	α,β-unsaturated ester	N-phosphine-oxazoline
1	1	Ir	tri-sub	aryl-alkyl	phosphite-oxazoline
1	1	Ir	tri-sub	aryl-alkyl	N-phosphine-thiazole
1	1	Ir	tri-sub	aryl-alkyl-N	phosphinite-pyridine
1	1	Ir	tri-sub	aryl-alkyl-N	ferrocene-phosphine-N-donor
1	1	Ir	tri-sub	aryl-alkyl-N	N-phosphine-oxazoline
1	1	Ir	tri-sub	α,β-unsaturated ester	phosphinite-pyridine
1	1	Ir	di-sub	α,β-unsaturated ester	phosphine-carbene
1	1	Ir	tri-sub	α,β-unsaturated ester	phosphine-O
1	1	Ir	tri-sub	α,β-unsaturated ester	phosphinite-pyridine
1	1	Ir	tri-sub	α,β-unsaturated ester	phosphinite-pyridine
1	1	Ir	tri-sub	α,β-unsaturated ketone	phosphine-oxazoline
1	1	Ir	tri-sub	α,β-unsaturated ester	phosphite-pyridine
1	1	Ir	tri-sub	aryl-alkyl	phosphine-oxazoline
1	1	Ir	tri-sub	aryl-alkyl	monophosphinite
1	1	Ir	tri-sub	sulphur	N-phosphine-thiazole
1	1	Ir	tri-sub	aryl-alkyl	phosphinite-oxazoline
1	1	Ir	tri-sub	aryl-alkyl	phosphinite-oxazoline
1	1	Ir	tri-sub	α,β-unsaturated ester	phosphinite-pyridine
1	1	Ir	tri-sub	allyl alcohol	phosphine-oxazoline
1	1	Ir	tri-sub	α,β-unsaturated ester	phosphine-O
1	1	Ir	tri-sub	boron	phosphite-pyridine
1	1	Ir	tri-sub	aryl-alkyl	phosphine-pyridine
1	1	Ir	tri-sub	aryl-alkyl-N	phosphine-O

1	1	Ir	tri-sub	aryl-alkyl-N	phosphine-oxazoline
1	1	Ir	tri-sub	allyl alcohol	phoshite-pyridine
1	1	Ir	tri-sub	α,β-unsaturated ketone	phosphite-oxazoline
1	1	Ir	tri-sub	α,β-unsaturated ester	phosphoramidite-oxazoline
1	1	Ir	tri-sub	α,β-unsaturated ester	phosphoramidite-oxazoline
1	1	Ir	tri-sub	aryl-alkyl	phosphine-oxazoline
1	1	Ir	tri-sub	α,β-unsaturated ester	phosphoramidite-pyridine
1	1	Ir	tri-sub	aryl-alkyl	phosphite-pyridine
1	1	Ir	tri-sub	aryl-alkyl-N	phosphine-thiazole
1	1	Ir	tri-sub	aryl-alkyl	phosphoramidite-oxazoline
1	1	Ir	tri-sub	α,β-unsaturated ester	phosphine-oxazoline
1	1	Ir	tri-sub	allyl alcohol	phosphinite-pyridine
1	1	Ir	tri-sub	enol	phosphinite-pyridine
1	1	Ir	tri-sub	α,β-unsaturated ester	phosphinite-pyridine
1	1	Ir	tri-sub	α,β-unsaturated ester	phosphite-oxazoline
1	1	Ir	tri-sub	aryl-alkyl	phosphite-pyridine
1	1	Ir	tri-sub	boron	phosphite-pyridine
1	1	Ir	tri-sub	aryl-alkyl	phosphite-pyridine
1	1	Ir	tri-sub	aryl-alkyl	phosphine-thiazole
1	1	Ir	tri-sub	α,β-unsaturated ester	phosphine-O
1	1	Ir	tri-sub	α,β-unsaturated ester	phosphinite-pyridine
1	1	Ir	tri-sub	aryl-alkyl	phosphoramidite-oxazoline
1	1	Ir	tri-sub	aryl-alkyl	phosphinite-pyridine
1	1	Ir	tri-sub	α,β-unsaturated ester	phosphoramidite-pyridine
1	1	Ir	tri-sub	aryl-alkyl-N	phosphine-thiazole
1	1	Ir	tri-sub	α,β-unsaturated ketone	phosphite-thiazole
1	1	Ir	di-sub	α,β-unsaturated acid	phosphine-N-donor
1	1	Ir	di-sub	enamide	phosphine-N-donor
1	1	Ir	di-sub	phosphorous	phosphite-oxazoline
1	1	Ir	tri-sub	aryl-alkyl	phosphine-imidazole
1	1	Ir	tri-sub	enol ether	phosphine-imidazole
1	1	Ir	tri-sub	aryl-alkyl	N-phosphine-thiazole
1	1	Ir	di-sub	enamide	phosphine-N-donor
1	1	Ir	tri-sub	enamine	phosphoramidite
1	1	Ir	tri-sub	α,β-unsaturated amide	ferrocene-phosphine-oxazoline

1	1	Ir	tri-sub	α,β-unsaturated ketone	phosphite-oxazoline
1	1	Ir	tri-sub	α,β-unsaturated ester	phosphine-N-donor
1	1	Ir	tri-sub	allyl ether	N-phosphine-thiazole
1	1	Ir	di-sub	enamide	phosphine-N-donor
1	1	Ir	tri-sub	aryl-alkyl	monophosphinite
1	1	Ir	tri-sub	aryl-alkyl	phosphinite-oxazoline
1	1	Ir	tri-sub	allyl ester	phosphite-thiazole
1	1	Ir	di-sub	allyl alcohol	phosphite-thiazole
1	1	Ir	tri-sub	allyl alcohol	phosphine-thiazole
1	1	Ir	tri-sub	aryl-alkyl	phosphine-thiazole
1	1	Ir	tetra-sub	α,β-unsaturated ester	N-phosphine-oxazoline
1	1	Ir	tri-sub	aryl-alkyl	monophosphinite
1	1	Ir	tri-sub	aryl-alkyl	monophosphinite
1	1	Ir	tri-sub	aryl-alkyl	phosphine-imidazole
1	1	Ir	tri-sub	aryl-alkyl-N	phosphine-thiazole
1	1	Ir	tri-sub	allyl ether	phosphine-thiazole
1	1	Ir	tri-sub	aryl-alkyl	phosphine-imidazole
1	1	Ir	tetra-sub	aryl-alkyl	phosphinite-oxazoline
1	1	Ir	di-sub	phosphorous	N-phosphine-thiazole
1	1	Ir	di-sub	enamide	phosphine-N-donor
1	1	Ir	tri-sub	aryl-alkyl	phosphine-thiazole
1	1	Ir	di-sub	phosphorous	N-phosphine-thiazole
1	1	Ir	tri-sub	α,β-unsaturated ketone	phosphite-thiazole
1	1	Ir	tri-sub	aryl-alkyl-N	ferrocene phosphinite-oxazoline
1	1	Ir	tri-sub	aryl-alkyl	N-phosphine-thiazole
1	1	Ir	tri-sub	enamine	phosphoramidite
1	1	Ir	tri-sub	aryl-alkyl	phosphite-thioether
1	1	Ir	tetra-sub	α,β-unsaturated ester	N-phosphine-oxazoline
1	1	Ir	tri-sub	α,β-unsaturated ester	phosphoramidite-oxazoline
1	1	Ir	tri-sub	aryl-alkyl-N	phosphine-N-donor
1	1	Ir	tri-sub	aryl-alkyl	phosphite-oxazoline
1	1	Ir	tri-sub	aryl-alkyl	phosphoramidite-oxazoline
1	1	Ir	di-sub	aryl-alkyl	phosphine-oxazoline
1	1	Ir	tetra-sub	aryl-alkyl	phosphinite-oxazoline
1	1	Ir	tetra-sub	aryl-alkyl	N-phosphine-oxazoline
1	1	Ir	tetra-sub	aryl-alkyl	phosphinite-oxazoline
1	1	Ir	di-sub	aryl-alkyl	phosphine-oxazoline
1	1	Ir	tetra-sub	aryl-alkyl	phosphine-oxazoline
1	1	Ir	di-sub	aryl-alkyl	phosphine-oxazoline
1	1	Ir	tri-sub	aryl-alkyl-N	phosphite-oxazoline

1	1	Ir	tri-sub	α,β-unsaturated ketone	bisphosphine
1	1	Ir	tri-sub	α,β-unsaturated amide	phosphine-oxazoline
1	1	Ir	tri-sub	α,β-unsaturated amide	phosphine-oxazoline
1	1	Ir	tetra-sub	aryl-alkyl	phosphine-imidazole
1	1	Ir	tri-sub	α,β-unsaturated amide	ferrocene phosphine-oxazoline
1	1	Ir	tri-sub	α,β-unsaturated amide	ferrocene phosphine-oxazoline
1	1	Ir	tetra-sub	aryl-alkyl	phosphinite-oxazoline
1	1	Ir	tri-sub	α,β-unsaturated ester	phosphinite-pyridine
1	1	Ir	tri-sub	α,β-unsaturated ketone	bisphosphine
1	1	Ir	tri-sub	α,β-unsaturated amide	ferrocene phosphine-oxazoline
1	1	Ir	tri-sub	α,β-unsaturated amide	phosphine-oxazoline
1	1	Ir	tri-sub	allyl ether	phosphine-oxazoline
1	1	Ir	tri-sub	α,β-unsaturated amide	ferrocene phosphine-oxazoline
1	1	Ir	tri-sub	aryl-alkyl-N	phoshite-oxazoline
1	1	Ir	tri-sub	α,β-unsaturated ketone	phosphine-oxazoline
1	1	Ir	tri-sub	α,β-unsaturated ketone	phosphine-oxazoline
1	1	Ir	tri-sub	α,β-unsaturated acid	phosphine-oxazoline
1	1	Ir	tri-sub	aryl-alkyl	monophosphinite
1	1	Ir	tetra-sub	α,β-unsaturated ester	N-phosphine-oxazoline
1	1	Ir	tri-sub	α,β-unsaturated acid	phosphine-oxazoline
1	1	Ir	tetra-sub	α,β-unsaturated ester	N-phosphine-oxazoline
1	1	Ir	tri-sub	α,β-unsaturated acid	phosphine-oxazoline
1	1	Ir	tri-sub	α,β-unsaturated amide	phosphine-oxazoline
1	1	Ir	tri-sub	α,β-unsaturated acid	phosphine-oxazoline
1	1	Ir	tri-sub	α,β-unsaturated acid	phosphine-oxazoline
1	1	Ir	tri-sub	α,β-unsaturated amide	phosphine-oxazoline
1	1	Ir	tri-sub	α,β-unsaturated acid	phosphine-oxazoline

1	1	Ir	di-sub	aryl-alkyl	phosphinite-oxazoline
1	1	Ir	tri-sub	α,β-unsaturated amide	phosphine-oxazoline
1	1	Ir	tri-sub	α,β-unsaturated amide	phosphine-oxazoline
1	1	Ir	tri-sub	α,β-unsaturated amide	phosphine-oxazoline
1	1	Ir	tri-sub	aryl-alkyl	monophosphinite
1	1	Ir	tri-sub	α,β-unsaturated amide	phosphine-oxazoline
1	1	Ir	tri-sub	α,β-unsaturated ketone	phosphine-oxazoline
1	1	Ir	tri-sub	α,β-unsaturated amide	phosphine-oxazoline
1	1	Ir	tri-sub	α,β-unsaturated amide	phosphine-oxazoline
1	1	Ir	tetra-sub	allyl alcohol	N-phosphine-oxazoline
1	1	Ir	tetra-sub	α,β-unsaturated ester	N-phosphine-oxazoline
1	1	Ir	tri-sub	aryl-alkyl	phosphite-thiazole
1	1	Ir	tri-sub	aryl-alkyl	phosphite-thiazole
1	1	Ir	tri-sub	α,β-unsaturated acid	phosphine-oxazoline
1	1	Ir	tri-sub	α,β-unsaturated ester	carbene-oxazoline
1	1	Ir	di-sub	boron	phosphinite-imidazole
1	1	Ir	tri-sub	α,β-unsaturated amide	bisphosphine
1	1	Ir	tri-sub	α,β-unsaturated acid	phosphine-oxazoline
1	1	Ir	tri-sub	boron	phoshine-oxazoline
1	1	Ir	di-sub	enol phosphinate	N-phosphine-oxazoline
1	1	Ir	tri-sub	aryl-alkyl-N	phosphite-thiazole
1	1	Ir	di-sub	aryl-alkyl	phosphite-pyridine
1	1	Ir	tri-sub	aryl-alkyl-N	phosphine-oxazoline
1	1	Ir	di-sub	aryl-alkyl	phosphite-pyridine
1	1	Ir	di-sub	enol phosphinate	N-phosphine-oxazoline
1	1	Ir	tri-sub	α,β-unsaturated acid	phosphine-oxazoline
1	1	Ir	di-sub	aryl-alkyl	phoshite-oxazoline
1	1	Ir	di-sub	aryl-alkyl(COOH)	phosphine-oxazoline
1	1	Ir	tri-sub	α,β-unsaturated acid	phosphine-oxazoline
1	1	Ir	tri-sub	α,β-unsaturated acid	phosphine-oxazoline
1	1	Ir	di-sub	aryl-alkyl(COOH)	phosphine-oxazoline
1	1	Ir	di-sub	aryl-alkyl	phosphite-thioether

1	1	Ir	tri-sub	α,β-unsaturated acid	phosphine-oxazoline
1	1	Ir	tri-sub	aryl-alkyl	phosphine-oxazoline
1	1	Ir	tri-sub	aryl-alkyl-N	phosphine-oxazoline
1	1	Ir	di-sub	aryl-alkyl	phosphite-pyridine
1	1	Ir	tri-sub	α,β-unsaturated amide	bisphosphine
1	1	Ir	di-sub	enol phosphinate	N-phosphine-oxazoline
1	1	Ir	tri-sub	α,β-unsaturated acid	phosphine-oxazoline
1	1	Ir	di-sub	aryl-alkyl	phoshite-oxazoline
1	1	Ir	tri-sub	aryl-alkyl	phosphite-thiazole
1	1	Ir	tri-sub	aryl-alkyl	phosphite-thiazole
1	1	Ir	tri-sub	α,β-unsaturated acid	phosphine-oxazoline
1	1	Ir	di-sub	enol phosphinate	N-phosphine-oxazoline
1	1	Ir	di-sub	aryl-alkyl	phosphite-thiazole
1	1	Ir	tri-sub	aryl-alkyl	phosphite-thiazole
1	1	Ir	di-sub	aryl-alkyl	phosphite-thiazole
1	1	Ir	tri-sub	α,β-unsaturated ester	phosphite-oxazoline
1	1	Ir	tri-sub	aryl-alkyl-N	phosphite-oxazoline
1	1	Ir	tri-sub	aryl-alkyl	phosphite-oxazoline
1	1	Ir	tri-sub	α,β-unsaturated acid	phosphine-oxazoline
1	1	Ir	tri-sub	α,β-unsaturated acid	phosphine-oxazoline
1	1	Ir	tri-sub	aryl-alkyl	phosphite-thiazole
1	1	Ir	di-sub	aryl-alkyl	phosphite-oxazoline
1	1	Ir	di-sub	aryl-alkyl	phoshite-oxazoline
1	1	Ir	tri-sub	α,β-unsaturated acid	phosphine-oxazoline
1	1	Ir	di-sub	aryl-alkyl	phosphite-oxazoline
1	1	Ir	di-sub	aryl-alkyl	phosphine-oxazoline
1	1	Ir	di-sub	aryl-alkyl	phosphite-pyridine
1	1	Ir	di-sub	aryl-alkyl	phosphite-thiazole
1	1	Ir	tri-sub	α,β-unsaturated acid	phosphine-oxazoline
1	1	Ir	tri-sub	aryl-alkyl	phosphine-oxazoline
1	1	Ir	tri-sub	α,β-unsaturated acid	phosphine-oxazoline
1	1	Ir	di-sub	aryl-alkyl	phosphite-thiazole
1	1	Ir	di-sub	aryl-alkyl	phoshite-oxazoline
1	1	Rh	tri-sub	α,β-unsaturated ketone	ferrocene bisphosphine
1	1	Rh	tri-sub	enamide	bisphosphine
1	1	Rh	tetra-sub	enamide	ferrocene bisphosphine

1	1	Rh	tri-sub	α,β-unsaturated ester	ferrocene bisphosphine
1	1	Rh	tri-sub	α,β-unsaturated ester	bisphosphine
1	1	Rh	tri-sub	α,β-unsaturated amide	ferrocene bisphosphine
1	1	Rh	tri-sub	enamide	bisphosphine
1	1	Rh	tri-sub	sulphur(SO2)	ferrocene bisphosphine
1	1	Rh	tri-sub	α,β-unsaturated ketone	ferrocene bisphosphine
1	1	Rh	tri-sub	α,β-unsaturated ketone	ferrocene bisphosphine
1	1	Rh	tri-sub	α,β-unsaturated ketone	ferrocene bisphosphine
1	1	Rh	tri-sub	α,β-unsaturated ester	ferrocene bisphosphine
1	1	Rh	tri-sub	α,β-unsaturated ester	ferrocene bisphosphine
1	1	Rh	tri-sub	sulphur(SO2)	ferrocene bisphosphine
1	1	Rh	tri-sub	α,β-unsaturated ester	ferrocene bisphosphine
1	1	Rh	tri-sub	α,β-unsaturated ester	bisphosphine
1	1	Rh	tetra-sub	enamide	ferrocene bisphosphine
1	1	Rh	tri-sub	α,β-unsaturated ester	ferrocene bisphosphine
1	1	Rh	tri-sub	enamide	phosphite-phosphine
1	1	Rh	tri-sub	sulphur(SO2)	ferrocene bisphosphine
1	1	Rh	tri-sub	α,β-unsaturated amide	ferrocene bisphosphine
1	1	Rh	tri-sub	α,β-unsaturated ester	ferrocene bisphosphine
1	1	Rh	tri-sub	α,β-unsaturated ester	ferrocene bisphosphine
1	1	Rh	tri-sub	α,β-unsaturated ester	ferrocene bisphosphine
1	1	Rh	tri-sub	α,β-unsaturated ester	ferrocene bisphosphine
1	1	Rh	tetra-sub	enamide	ferrocene bisphosphine
1	1	Rh	tri-sub	α,β-unsaturated ester	ferrocene bisphosphine
1	1	Rh	tri-sub	nitro	ferrocene bisphosphine
1	1	Rh	tri-sub	sulphur(SO2)	ferrocene bisphosphine
1	1	Rh	tri-sub	α,β-unsaturated ester	ferrocene bisphosphine
1	1	Rh	tri-sub	α,β-unsaturated ester	ferrocene bisphosphine
1	1	Rh	tri-sub	α,β-unsaturated ester	ferrocene bisphosphine

1	1	Rh	tri-sub	α,β-unsaturated amide	ferrocene bisphosphine
1	1	Rh	tri-sub	sulphur(SO2)	ferrocene bisphosphine
1	1	Rh	tri-sub	α,β-unsaturated ester	ferrocene bisphosphine
1	1	Rh	tri-sub	α,β-unsaturated amide	ferrocene bisphosphine
1	1	Rh	tri-sub	α,β-unsaturated ester	ferrocene bisphosphine
1	1	Rh	tri-sub	α,β-unsaturated ester	ferrocene bisphosphine
1	1	Rh	tri-sub	α,β-unsaturated amide	ferrocene bisphosphine
1	1	Rh	tri-sub	α,β-unsaturated ester	ferrocene bisphosphine
1	1	Rh	tetra-sub	enamide	ferrocene bisphosphine
1	1	Rh	tri-sub	sulphur(SO2)	ferrocene bisphosphine
1	1	Rh	di-sub	α,β-unsaturated ester	phosphoramidite
1	1	Rh	tri-sub	phosphorous	phosphite-phosphine
1	1	Rh	tri-sub	enamide	bisphosphine
1	1	Rh	tri-sub	enamide	phosphoramidite
1	1	Rh	tri-sub	enamide	phosphoramidite-phosphoramidite
1	1	Rh	di-sub	α,β-unsaturated ester	phosphite-phosphine
1	1	Rh	tri-sub	enamide	phosphoramidite
1	1	Rh	tri-sub	enamide	bisphosphine
1	1	Rh	di-sub	enamide	bisphosphine
1	1	Rh	tri-sub	enamide	phosphite
1	1	Rh	tri-sub	enamide	phosphoramidite-phosphine
1	1	Rh	tri-sub	enamide	phosphoramidite-phosphoramidite
1	1	Rh	di-sub	aryl-alkyl (Py)	bisphosphine
1	1	Rh	tri-sub	enamide	phosphoramidite
1	1	Rh	tri-sub	enamide	bisphosphine
1	1	Rh	di-sub	α,β-unsaturated ester	phosphite
1	1	Rh	di-sub	sulphur(SO2)	bisphosphine
1	1	Rh	di-sub	enamide	phosphoramidite-phosphine-ferrocene
1	1	Rh	tri-sub	enamide	phosphoramidite
1	1	Rh	tri-sub	α,β-unsaturated ester	phosphoramidite
1	1	Rh	di-sub	α,β-unsaturated acid	bisphosphine
1	1	Rh	tri-sub	enamide	phosphoramidite-phosphoramidite

1	1	Rh	di-sub	phosphorous	bisphosphine
1	1	Rh	di-sub	enamide	phosphite
1	1	Rh	di-sub	α,β-unsaturated acid	ferrocene phosphine-N-donor
1	1	Rh	tri-sub	enamide	bisphosphine
1	1	Rh	tri-sub	enamide	phosphoramidite
1	1	Rh	di-sub	enamide	phoshite
1	1	Rh	tri-sub	enamide	ferrocene bisphosphine
1	1	Rh	tri-sub	α,β-unsaturated ester	bisphosphine
1	1	Rh	di-sub	α,β-unsaturated ketone	bisphosphine
1	1	Rh	tri-sub	enamide	phosphite
1	1	Rh	tri-sub	enamide	phosphoramidite
1	1	Rh	tri-sub	enamide	phoshite
1	1	Rh	tri-sub	enamide	bisphosphine
1	1	Rh	di-sub	α,β-unsaturated ester	bisphosphine
1	1	Rh	tri-sub	enamide	ferrocene bisphosphine
1	1	Rh	di-sub	α,β-unsaturated acid	phosphoramidite
1	1	Rh	tri-sub	enamide	bisphosphine
1	1	Rh	tri-sub	enamide	phosphoramidite
1	1	Rh	di-sub	α,β-unsaturated acid	bisphosphine
1	1	Rh	tri-sub	enamide	bisphosphine
1	1	Rh	tri-sub	enamide	ferrocene bisphosphine
1	1	Rh	di-sub	enamide	phosphoramidite-ferrocene
1	1	Rh	di-sub	α,β-unsaturated acid	ferrocene phosphine-N-donor
1	1	Rh	di-sub	enamide	phosphite
1	1	Rh	tri-sub	enamide	bisphosphine
1	1	Rh	tri-sub	enamide	phosphoramidite-phosphine-ferrocene
1	1	Rh	di-sub	enamide	phosphoramidite
1	1	Rh	di-sub	enamide	bisphosphine
1	1	Rh	tri-sub	enamide	phosphoramidite
1	1	Rh	di-sub	α,β-unsaturated ester	phosphoramidite
1	1	Rh	tri-sub	enamide	amino phosphines
1	1	Rh	tri-sub	enamide	phosphinite
1	1	Rh	di-sub	enamide	bisphosphine
1	1	Rh	tri-sub	enamide	phosphoramidite-phosphoramidite
1	1	Rh	tri-sub	enamide	ferrocene bisphosphine
1	1	Rh	di-sub	phosphorous	ferrocene bisphosphine
1	1	Rh	tri-sub	enamide	bisphosphine

1	1	Rh	tri-sub	α,β-unsaturated ester	phoshite
1	1	Rh	tri-sub	α,β-unsaturated ester	phosphite
1	1	Rh	tri-sub	enamide	bisphosphine
1	1	Rh	tri-sub	enamide	bisphosphine
1	1	Rh	tri-sub	α,β-unsaturated amide	ferrocene bisphosphine
1	1	Rh	tri-sub	enamide	biphosphinite
1	1	Rh	di-sub	α,β-unsaturated ester	bisphosphine
1	1	Rh	di-sub	enamide	bisphosphine
1	1	Rh	tri-sub	enamide	phosphoramidite
1	1	Rh	di-sub	α,β-unsaturated ester	phosphoramidite-phosphine-ferrocene
1	1	Rh	di-sub	enamide	bisphosphine
1	1	Rh	di-sub	α,β-unsaturated ester	phosphoramidite-phosphoramidite
1	1	Rh	tri-sub	enamide	bisphosphine
1	1	Rh	tri-sub	enamide	bisphosphine
1	1	Rh	tri-sub	α,β-unsaturated amide	ferrocene bisphosphine
1	1	Rh	di-sub	α,β-unsaturated ester	phosphoramidite
1	1	Rh	tri-sub	α,β-unsaturated acid	ferrocene-phosphine-N-donor
1	1	Rh	di-sub	α,β-unsaturated ester	phosphite
1	1	Rh	di-sub	α,β-unsaturated ester	phosphoramidite- phosphite
1	1	Rh	tri-sub	enamide	bisphosphine
1	1	Rh	tri-sub	α,β-unsaturated acid	ferrocene bisphosphine
1	1	Rh	di-sub	α,β-unsaturated ester	phosphoramidite-phosphine-ferrocene
1	1	Rh	di-sub	enamide	phosphoramidite
1	1	Rh	tetra-sub	α,β-unsaturated ketone	ferrocene bisphosphine
1	1	Rh	tri-sub	enamide	bisphosphine
1	1	Rh	tri-sub	enamide	bisphosphine
1	1	Rh	tri-sub	enamide	bisphosphine
1	1	Rh	tetra-sub	α,β-unsaturated ester	ferrocene bisphosphine
1	1	Rh	di-sub	α,β-unsaturated ester	phosphoramidite-phosphoramidite
1	1	Rh	tri-sub	enamide	phosphoramidite
1	1	Rh	di-sub	enamide	bisphosphine
1	1	Rh	tri-sub	enamide	ferrocene bisphosphine

1	1	Rh	tri-sub	α,β-unsaturated ester	bisphosphine
1	1	Rh	tri-sub	enamide	bisphosphine
1	1	Rh	di-sub	enamide	bisphosphine
1	1	Rh	tri-sub	α,β-unsaturated ester	ferrocene bisphosphine
1	1	Rh	tetra-sub	α,β-unsaturated ketone	ferrocene bisphosphine
1	1	Rh	tri-sub	α,β-unsaturated acid	ferrocene-phosphine-N-donor
1	1	Rh	di-sub	enamide	bisphosphine
1	1	Rh	di-sub	enamide	phosphoramidite
1	1	Rh	tri-sub	α,β-unsaturated acid	ferrocene bisphosphine
1	1	Rh	tri-sub	α,β-unsaturated acid	ferrocene bisphosphine
1	1	Rh	di-sub	α,β-unsaturated ester	phosphite-phosphite
1	1	Rh	di-sub	enamide	bisphosphine
1	1	Rh	tri-sub	enamide	phosphoramidite
1	1	Rh	tetra-sub	enamide	ferrocene bisphosphine
1	1	Rh	tri-sub	aryl-alkyl-NO2	ferrocene bisphosphine
1	1	Rh	di-sub	enamide	phosphoramidite
1	1	Rh	tetra-sub	enamide	bisphosphine
1	1	Rh	tri-sub	α,β-unsaturated amide	ferrocene bisphosphine
1	1	Rh	tri-sub	aryl-alkyl-NO2	ferrocene bisphosphine
1	1	Rh	tri-sub	α,β-unsaturated amide	ferrocene bisphosphine
1	1	Rh	tri-sub	enamide	phosphoramidite
1	1	Rh	tri-sub	nitro	ferrocene bisphosphine
1	1	Rh	tri-sub	nitro	ferrocene bisphosphine
1	1	Rh	tetra-sub	enamide	ferrocene bisphosphine
1	1	Rh	tri-sub	α,β-unsaturated amide	ferrocene bisphosphine
1	1	Rh	tri-sub	α,β-unsaturated ester	ferrocene bisphosphine
1	1	Rh	tri-sub	α,β-unsaturated ester	ferrocene bisphosphine
1	1	Rh	tri-sub	α,β-unsaturated amide	ferrocene bisphosphine
1	1	Rh	tri-sub	α,β-unsaturated amide	ferrocene bisphosphine
1	1	Rh	tri-sub	enamide	bisphosphine
1	1	Rh	tri-sub	α,β-unsaturated amide	ferrocene bisphosphine
1	1	Rh	tetra-sub	enamide	bisphosphine

1	1	Rh	tri-sub	α,β-unsaturated amide	ferrocene bisphosphine
1	1	Rh	tri-sub	α,β-unsaturated ester	ferrocene bisphosphine
1	1	Rh	tri-sub	enamide	phosphoramidite
1	1	Rh	di-sub	α,β-unsaturated ester	phosphoramidite
1	1	Rh	tri-sub	enamide	ferrocene bisphosphine
1	1	Rh	tri-sub	enamide	phosphoramidite
1	1	Rh	tri-sub	α,β-unsaturated ester	ferrocene bisphosphine
1	1	Rh	tri-sub	enamide	phosphite
1	1	Rh	tri-sub	enamide	phosphite
1	1	Rh	tri-sub	enamide	phosphoramidite
1	1	Rh	tri-sub	α,β-unsaturated ester	phosphonite
1	1	Rh	tri-sub	enamide	phosphoramidite
1	1	Rh	tetra-sub	enamide	bisphosphine
1	1	Rh	di-sub	α,β-unsaturated ester	phosphoramidite
1	1	Rh	tri-sub	sulphur(SO2)	ferrocene bisphosphine
1	1	Rh	tri-sub	enamide	phosphoramidite
1	1	Rh	tri-sub	enamide	bisphosphine
1	1	Rh	di-sub	enamide	phosphoramidite
1	1	Rh	tri-sub	α,β-unsaturated amide	ferrocene bisphosphine
1	1	Rh	tri-sub	enamide	bisphosphine
1	1	Rh	tri-sub	α,β-unsaturated amide	ferrocene bisphosphine
1	1	Rh	di-sub	enamide	phosphoramidite
1	1	Rh	tri-sub	enamide	phosphoramidite
1	1	Rh	di-sub	enamide	phosphoramidite
1	1	Rh	tri-sub	enamide	phosphite
1	1	Rh	di-sub	enamide	phosphoramidite
1	1	Rh	tri-sub	enamide	phosphite
1	1	Rh	tetra-sub	enamide	bisphosphine
1	1	Rh	tri-sub	enamide	bisphosphine
1	1	Rh	di-sub	enamide	phosphoramidite
1	1	Rh	di-sub	enamide	phosphoramidite
1	1	Rh	di-sub	α,β-unsaturated ester	phosphite
1	1	Rh	di-sub	α,β-unsaturated ester	phosphoramidite
1	1	Rh	tri-sub	sulphur(SO2)	ferrocene bisphosphine
1	1	Rh	di-sub	enamide	phosphoramidite
1	1	Rh	tri-sub	α,β-unsaturated ketone	ferrocene bisphosphine

1	1	Rh	tri-sub	α,β-unsaturated ketone	ferrocene bisphosphine
1	1	Rh	di-sub	enamide	phosphoramidite
1	1	Rh	di-sub	enamide	phosphoramidite
1	1	Rh	tri-sub	enamide	phosphoramidite
1	1	Rh	di-sub	enamide	phosphoramidite
1	1	Rh	tri-sub	sulphur(SO2)	ferrocene bisphosphine
1	1	Rh	tri-sub	α,β-unsaturated acid	monodentate phosphine oxide
1	1	Rh	tri-sub	enamide	bisphosphine
1	1	Rh	tri-sub	enamide	phosphoramidite
1	1	Rh	tri-sub	α,β-unsaturated amide	ferrocene bisphosphine
1	1	Rh	tri-sub	phosphorous	phosphoramidite-ferrocene
1	1	Rh	tri-sub	enamide	phosphoramidite-phosphine-ferrocene
1	1	Rh	di-sub	enamide	phosphoramidite
1	1	Rh	tri-sub	α,β-unsaturated ester	ferrocene bisphosphine
1	1	Rh	tri-sub	α,β-unsaturated ester	ferrocene bisphosphine
1	1	Rh	tri-sub	enamide	phosphite-phosphine
1	1	Rh	tri-sub	enamide	phosphoramidite-phosphine-ferrocene
1	1	Rh	tri-sub	α,β-unsaturated acid	monodentate phosphine oxide
1	1	Rh	tetra-sub	enamide	bisphosphine
1	1	Rh	tri-sub	α,β-unsaturated amide	ferrocene bisphosphine
1	1	Rh	tri-sub	phosphorous	phosphoramidite-ferrocene
1	1	Rh	tri-sub	enamide	phosphoramidite
1	1	Rh	di-sub	enamide	phosphoramidite
1	1	Rh	di-sub	enol ester	phosphite-phosphine
1	1	Rh	tri-sub	enamide	phosphoramidite-phosphine-ferrocene
1	1	Rh	di-sub	α,β-unsaturated ester	phosphite-phosphine
1	1	Rh	di-sub	enamide	phosphoramidite
1	1	Rh	tri-sub	enamide	phosphoramidite
1	1	Rh	tri-sub	α,β-unsaturated acid	monodentate phosphine oxide
1	1	Rh	tri-sub	enamide	phosphoramidite
1	1	Rh	tri-sub	enamide	phosphoramidite-phosphine-ferrocene
1	1	Rh	tri-sub	phosphorous	phosphoramidite-ferrocene
1	1	Rh	tri-sub	enamide	phosphite-phosphine
1	1	Rh	di-sub	enamide	phosphite-phosphine

1	1	Rh	tri-sub	enamide	phosphoramidite-phosphoramidite
1	1	Rh	di-sub	enamide	phosphoramidite
1	1	Rh	tri-sub	enamide	phosphoramidite
1	1	Rh	tri-sub	enamide	phosphite-phosphine
1	1	Rh	tri-sub	enamide	biaryl phosphine
1	1	Rh	di-sub	α,β-unsaturated ester	phosphoramidite
1	1	Rh	tetra-sub	enamide	bisphosphine
1	1	Rh	tetra-sub	enamide	bisphosphine
1	1	Rh	tri-sub	enamide	phosphoramidite
1	1	Rh	tri-sub	enamide	phosphoramidite
1	1	Rh	di-sub	enamide	phosphoramidite
1	1	Rh	tri-sub	enamide	phosphonite
1	1	Rh	tri-sub	enamide	phosphoramidite
1	1	Rh	tri-sub	enamide	phosphoramidite
1	1	Rh	tri-sub	enamide	phosphoramidite-phosphine-ferrocene
1	1	Rh	tri-sub	enamide	phosphite
1	1	Rh	tri-sub	enamide	bisphosphine
1	1	Rh	di-sub	α,β-unsaturated acid	ferrocene-phosphine-N-donor
1	1	Rh	di-sub	phosphorous	ferrocene-phosphine-N-donor
1	1	Rh	tri-sub	enamide	phosphoramidite
1	1	Rh	di-sub	α,β-unsaturated acid	ferrocene-phosphine-N-donor
1	1	Rh	di-sub	α,β-unsaturated ester	phosphite
1	1	Rh	di-sub	enamide	phosphite
1	1	Rh	tri-sub	enamine	ferrocene bisphosphine
1	1	Rh	tri-sub	enamide	bisphosphine
1	1	Rh	di-sub	phosphorous	ferrocene-phosphine-N-donor
1	1	Rh	di-sub	α,β-unsaturated acid	ferrocene-phosphine-N-donor
1	1	Rh	tri-sub	enamine	ferrocene bisphosphine
1	1	Rh	di-sub	α,β-unsaturated ester	phosphite-phosphine
1	1	Rh	di-sub	α,β-unsaturated ester	phosphite
1	1	Rh	tri-sub	enamide	bisphosphine
1	1	Rh	di-sub	α,β-unsaturated acid	ferrocene-phosphine-N-donor
1	1	Rh	di-sub	α,β-unsaturated acid	ferrocene-phosphine-N-donor
1	1	Rh	di-sub	α,β-unsaturated acid	ferrocene-phosphine-N-donor

1	1	Rh	di-sub	phosphorous	ferrocene-phosphine-N-donor
1	1	Rh	tri-sub	enamide	bisphosphine
1	1	Rh	tri-sub	enamine	ferrocene bisphosphine
1	1	Rh	tri-sub	enamide	bisphosphine
1	1	Rh	tri-sub	enamine	ferrocene bisphosphine
1	1	Rh	di-sub	enamide	phosphoramidite
1	1	Rh	di-sub	enamide	phosphite
1	1	Rh	di-sub	α,β-unsaturated ester	phosphite
1	1	Rh	tri-sub	enamide	bisphosphine
1	1	Rh	tri-sub	enamide	phosphoramidite
1	1	Rh	di-sub	α,β-unsaturated ester	phosphite
1	1	Rh	tri-sub	enamide	bisphosphine
1	1	Rh	di-sub	α,β-unsaturated acid	ferrocene-phosphine-N-donor
1	0	Co	di-sub	silicon	phosphine-pyridine-oxazoline(PPO)
1	0	Co	tri-sub	α,β-unsaturated acid	bisphosphine
1	0	Ir	tetra-sub	α,β-unsaturated ester	ferrocene bisphosphine
1	0	Ir	di-sub	aryl-alkyl	phosphinite-oxazoline
1	0	Ir	tetra-sub	α,β-unsaturated ester	ferrocene bisphosphine
1	0	Ir	tetra-sub	α,β-unsaturated ester	ferrocene bisphosphine
1	0	Ir	tri-sub	α,β-unsaturated ester	N-phosphine-thiazole
1	0	Ir	tetra-sub	α,β-unsaturated ester	bisphosphine
1	0	Ir	di-sub	enamine	phosphoramidite
1	0	Ir	di-sub	aryl-alkyl	phosphinite-oxazoline
1	0	Ir	tri-sub	aryl-alkyl	phoshite-oxazoline
1	0	Ir	tri-sub	aryl-alkyl-N	phosphine-oxazoline
1	0	Ir	tri-sub	enol ether	phosphine-imidazole
1	0	Ir	tri-sub	aryl-alkyl-N	phosphine-carbene
1	0	Ir	tri-sub	aryl-alkyl	phosphine-imidazole
1	0	Ir	tri-sub	aryl-alkyl	phosphite-oxazoline
1	0	Ir	tri-sub	aryl-alkyl	phosphine-oxazoline
1	0	Ir	tri-sub	enol ether	phosphinite-oxazoline
1	0	Ir	tri-sub	aryl-alkyl	phosphite-pyridine
1	0	Ir	tri-sub	boron	phosphine-pyridine
1	0	Ir	tri-sub	α,β-unsaturated ester	phosphinite-pyridine
1	0	Ir	tri-sub	aryl-alkyl-N	ferrocene-phosphine-N-donor

1	0	Ir	tri-sub	aryl-alkyl	phosphine-oxazoline
1	0	Ir	di-sub	aryl-alkyl	phosphite-pyridine
1	0	Ir	di-sub	aryl-alkyl	N-phosphine-oxazoline
1	0	Ir	tri-sub	enamine	phosphoramidite
1	0	Ir	tri-sub	silicon	phosphinite-pyridine
1	0	Ir	di-sub	aryl-alkyl	phosphite-pyridine
1	0	Ir	tri-sub	aryl-alkyl	phosphine-thiazole
1	0	Ir	tri-sub	silicon	phosphinite-oxazoline
1	0	Ir	tri-sub	silicon	phosphite-thiazole
1	0	Ir	tri-sub	aryl-alkyl-N	phosphine-oxazoline
1	0	Ir	tri-sub	enamine	phosphoramidite
1	0	Ir	tri-sub	aryl-alkyl-N	phosphine-N-donor
1	0	Ir	tri-sub	α,β-unsaturated ester	phosphine-oxazoline
1	0	Ir	tri-sub	aryl-alkyl	phosphite-pyridine
1	0	Ir	tri-sub	aryl-alkyl	phosphite-thioether
1	0	Ir	tri-sub	α,β-unsaturated ester	phosphite-oxazoline
1	0	Ir	tetra-sub	aryl-alkyl	N-phosphine-oxazoline
1	0	Ir	tri-sub	α,β-unsaturated amide	phosphine-oxazoline
1	0	Ir	tri-sub	aryl-alkyl-N	N-phosphine-N-donor
1	0	Ir	tri-sub	aryl-alkyl-N	ferrocene-phosphine-N-donor
1	0	Ir	di-sub	α,β-unsaturated amide	phosphine-oxazoline
1	0	Ir	tetra-sub	aryl-alkyl	phosphinite-oxazoline
1	0	Ir	tri-sub	α,β-unsaturated acid	phosphine-oxazoline
1	0	Ir	tri-sub	α,β-unsaturated amide	phosphine-oxazoline
1	0	Ir	tri-sub	aryl-alkyl	phosphite-thiazole
1	0	Ir	tri-sub	aryl-alkyl-N	phoshite-oxazoline
1	0	Ir	di-sub	aryl-alkyl	phosphite-oxazoline
1	0	Ir	tri-sub	aryl-alkyl	phosphite-thiazole
1	0	Ir	tri-sub	silicon	phosphine-thiazole
1	0	Ir	di-sub	enamine	phosphinite-oxazoline
1	0	Ir	di-sub	silicon	phosphinite-pyridine
1	0	Ir	tetra-sub	α,β-unsaturated ester	bisphosphine
1	0	Ir	tri-sub	aryl-alkyl	phosphine-oxazoline
1	0	Ir	tri-sub	aryl-alkyl-N	ferrocene phosphine-oxazoline
1	0	Rh	tri-sub	enamide	ferrocene bisphosphine
1	0	Rh	tri-sub	enamide	bisphosphine
1	0	Rh	tri-sub	α,β-unsaturated ester	ferrocene bisphosphine

1	0	Rh	tri-sub	α,β-unsaturated ester	bisphosphine
1	0	Rh	tri-sub	α,β-unsaturated ester	ferrocene bisphosphine
1	0	Rh	tri-sub	enamide	bisphosphine
1	0	Rh	tri-sub	enamide	ferrocene bisphosphine
1	0	Rh	di-sub	α,β-unsaturated acid	ferrocene bisphosphine
1	0	Rh	tetra-sub	allyl alcohol	bisphosphine
1	0	Rh	di-sub	sulphur(SO2)	bisphosphine
1	0	Rh	di-sub	enamide	ferrocene bisphosphine
1	0	Rh	tri-sub	enamide	phosphine
1	0	Rh	tri-sub	enamide	phosphoramidite- phosphite
1	0	Rh	di-sub	enamide	phosphite- phosphoramidite
1	0	Rh	di-sub	α,β-unsaturated ester	phosphite
1	0	Rh	tri-sub	enamide	ferrocene bisphosphine
1	0	Rh	di-sub	α,β-unsaturated acid	phosphoramidite
1	0	Rh	di-sub	enamide	ferrocene bisphosphine
1	0	Rh	tri-sub	enamide	ferrocene bisphosphine
1	0	Rh	di-sub	enamide	bisphosphine
1	0	Rh	tri-sub	enamide	phosphoramidite-phosphine
1	0	Rh	di-sub	α,β-unsaturated ester	phosphoramidite-phosphine-ferrocene
1	0	Rh	di-sub	enamide	bisphosphine
1	0	Rh	di-sub	α,β-unsaturated ester	phosphonite
1	0	Rh	tetra-sub	α,β-unsaturated ketone	ferrocene bisphosphine
1	0	Rh	di-sub	enamide	phosphoramidite-N donor
1	0	Rh	tri-sub	enamide	phosphoramidite
1	0	Rh	tri-sub	enamide	phosphoramidite
1	0	Rh	tetra-sub	enamide	bisphosphine
1	0	Rh	tri-sub	enamide	phosphoramidite
1	0	Rh	tri-sub	enamide	phosphoramidite
1	0	Rh	tri-sub	enamide	phosphoramidite
1	0	Rh	tri-sub	enamide	phosphite
1	0	Rh	tri-sub	enamide	phosphoramidite
1	0	Rh	tri-sub	enamide	biaryl phosphine
1	0	Rh	tri-sub	enamide	phosphoramidite
1	0	Rh	tri-sub	enamide	phoshite
1	0	Rh	tri-sub	enamide	phosphite-phosphine
1	0	Rh	di-sub	boron	ferrocene bisphosphine
1	0	Rh	tri-sub	α,β-unsaturated acid	monodentate phosphine oxide
1	0	Rh	tri-sub	α,β-unsaturated acid	monodentate phosphine oxide

1	0	Rh	di-sub	enol carbamate	biaryl phosphine
1	0	Rh	tetra-sub	enamide	bisphosphine
1	0	Rh	tri-sub	enamide	ferrocene bisphosphine
1	0	Rh	di-sub	α,β-unsaturated ester	phosphoramidite
1	0	Rh	di-sub	enamide	phosphite
1	0	Rh	tri-sub	enamide	bisphosphine
1	0	Rh	tri-sub	enamide	bisphosphine
1	0	Rh	tetra-sub	enamide	bisphosphine
1	0	Rh	di-sub	enol ester	phosphite
1	0	Rh	di-sub	α,β-unsaturated ester	phosphoramidite
1	0	Rh	di-sub	α,β-unsaturated ester	phosphoramidite
0	1	Co	di-sub	aryl-alkyl-N	bisphosphine
0	1	Co	tri-sub	enamide	bisphosphine
0	1	Co	di-sub	silicon	phosphine-pyridine-oxazoline(PPO)
0	1	Co	tri-sub	aryl-alkyl	bisphosphine
0	1	Co	di-sub	aryl-alkyl	NNN-pincer
0	1	Co	di-sub	aryl-alkyl	NNN-pincer
0	1	Co	di-sub	silicon	phosphine-pyridine-oxazoline(PPO)
0	1	Co	tri-sub	enamide	bisphosphine
0	1	Ir	di-sub	α,β-unsaturated acid	phosphine-O
0	1	Ir	tri-sub	aryl-alkyl	phosphine-thiazole
0	1	Ir	tetra-sub	aryl-alkyl	N-phosphine-oxazoline
0	1	Ir	di-sub	aryl-alkyl	carbene-pyridine
0	1	Ir	tri-sub	aryl-alkyl	phosphine-oxazoline
0	1	Ir	di-sub	aryl-alkyl	carbene-pyridine
0	1	Ir	di-sub	aryl-alkyl	phosphite-oxazoline
0	1	Ir	tri-sub	α,β-unsaturated ester	phosphine-oxazoline
0	1	Ir	tri-sub	aryl-alkyl	phosphine-oxazoline
0	1	Ir	tri-sub	allyl ester	phosphine-pyridine
0	1	Ir	tri-sub	α,β-unsaturated ester	phosphinite-pyridine
0	1	Ir	tri-sub	allyl alcohol	phosphinite-pyridine
0	1	Ir	tri-sub	α,β-unsaturated ester	phosphinite-oxazoline
0	1	Ir	tri-sub	allyl alcohol	phosphinite-pyridine
0	1	Ir	tri-sub	α,β-unsaturated ester	phosphine-oxazoline
0	1	Ir	tri-sub	aryl-alkyl	phosphite-pyridine
0	1	Ir	tri-sub	α,β-unsaturated amide	phosphine-oxazoline
0	1	Ir	tri-sub	aryl-alkyl	phosphine-imidazole

0	1	Ir	di-sub	aryl-alkyl	N-phosphine-oxazoline
0	1	Ir	di-sub	phosphorous	N-phosphine-oxazoline
0	1	Ir	tri-sub	aryl-alkyl-N	phosphine-N-donor
0	1	Ir	tri-sub	aryl-alkyl-N	phosphoramidite-oxazoline
0	1	Ir	tri-sub	aryl-alkyl	phosphoramidite-oxazoline
0	1	Ir	tri-sub	boron	ferrocene-phosphine-N-donor
0	1	Ir	tri-sub	α,β-unsaturated ketone	bisphosphine
0	1	Ir	tri-sub	α,β-unsaturated ester	phosphine-oxazoline
0	1	Ir	tri-sub	α,β-unsaturated ester	phosphine-oxazoline
0	1	Ir	tri-sub	aryl-alkyl-N	phoshite-oxazoline
0	1	Ir	tri-sub	α,β-unsaturated amide	phosphine-oxazoline
0	1	Ir	di-sub	aryl-alkyl	phosphite-thioether
0	1	Ir	tetra-sub	aryl-alkyl	phosphine-imidazole
0	1	Ir	tri-sub	enamine	phosphoramidite
0	1	Ir	tri-sub	aryl-alkyl	phosphine-oxazoline
0	1	Ir	tri-sub	aryl-alkyl	phosphite-thiazole
0	1	Ir	tri-sub	α,β-unsaturated amide	bisphosphine
0	1	Ir	tri-sub	allyl alcohol	phosphinite-oxazoline
0	1	Ir	tri-sub	aryl-alkyl	phosphite-thiazole
0	1	Ir	di-sub	enol phosphinate	phosphinite-oxazoline
0	1	Ir	di-sub	boron	phosphinite-imidazole
0	1	Ir	tri-sub	enamide	phosphine-oxazoline
0	1	Ir	di-sub	silicon	phosphinite-oxazoline
0	1	Rh	tri-sub	enamide	ferrocene bisphosphine
0	1	Rh	tetra-sub	α,β-unsaturated ester	ferrocene bisphosphine
0	1	Rh	tetra-sub	α,β-unsaturated ester	ferrocene bisphosphine
0	1	Rh	tri-sub	enamide	bisphosphine
0	1	Rh	tri-sub	α,β-unsaturated amide	ferrocene bisphosphine
0	1	Rh	tri-sub	α,β-unsaturated ester	ferrocene bisphosphine
0	1	Rh	di-sub	enamide	phosphite
0	1	Rh	tri-sub	enamide	bisphosphine
0	1	Rh	di-sub	enamide	phosphoramidite
0	1	Rh	di-sub	α,β-unsaturated acid	ferrocene bisphosphine
0	1	Rh	di-sub	α,β-unsaturated ester	phosphoramidite
0	1	Rh	di-sub	α,β-unsaturated ester	phosphite

0	1	Rh	tri-sub	enamide	bisphosphine
0	1	Rh	tri-sub	enamide	ferrocene bisphosphine
0	1	Rh	tri-sub	sulphur(SO2)	ferrocene bisphosphine
0	1	Rh	tri-sub	enamide	bisphosphine
0	1	Rh	di-sub	enamide	phosphite
0	1	Rh	di-sub	α,β-unsaturated ester	phosphite- phosphite
0	1	Rh	tri-sub	α,β-unsaturated amide	ferrocene bisphosphine
0	1	Rh	tri-sub	enamide	phosphoramidite
0	1	Rh	tri-sub	α,β-unsaturated amide	ferrocene bisphosphine
0	1	Rh	tri-sub	α,β-unsaturated ester	ferrocene bisphosphine
0	1	Rh	tri-sub	enamide	phosphoramidite
0	1	Rh	tri-sub	enamide	phosphonite
0	1	Rh	tri-sub	enamide	phosphonite
0	1	Rh	tri-sub	enamide	phosphoramidite
0	1	Rh	tri-sub	enamide	phosphoramidite
0	1	Rh	tri-sub	enamide	phosphite
0	1	Rh	tri-sub	enamide	phosphite-phosphine
0	1	Rh	di-sub	enol ester	phosphite-phosphine
0	1	Rh	di-sub	α,β-unsaturated acid	phosphine
0	1	Rh	tri-sub	enamide	biaryl phosphine
0	1	Rh	tetra-sub	enamide	bisphosphine
0	1	Rh	tri-sub	enamide	bisphosphine
0	1	Rh	di-sub	boron	ferrocene bisphosphine
0	1	Rh	tri-sub	enamine	ferrocene bisphosphine
0	1	Rh	tri-sub	enamine	ferrocene bisphosphine
0	1	Rh	tri-sub	α,β-unsaturated acid	ferrocene bisphosphine
0	1	Rh	di-sub	enamide	phosphoramidite
0	1	Rh	di-sub	enamide	phosphite
0	1	Rh	tetra-sub	α,β-unsaturated ketone	ferrocene bisphosphine

8. Effect of clustering on performance of meta-cluster model

To show that the process of clustering (section 4) has no significant effect on the model performance, we repeated it ten times. The performance of these ten runs is shown in Table S15. Since we obtained the best AUPRC score of 0.9300±0.0011 with full cluster data as

support set during test time (Table S8), we use this for further analysis. In addition, the model performance with different cluster sized (k=10, 15, and 20) is reported in Table S15.

Table S15. Model Performance with Different Number of Clusters and 10 Runs

Number of clusters = 10		
No. of Runs	AUROC	AUPRC
1	0.8932±0.0017	0.9279±0.0027
2	0.8913±0.0016	0.9222±0.0009
3	0.8870±0.0024	0.9184±0.0024
4	0.8862±0.0025	0.9215±0.0041
5	0.8863±0.0012	0.9185±0.0019
6	0.8900±0.0020	0.9245±0.0021
7	0.8911±0.0035	0.9254±0.0022
8	0.8879±0.0027	0.9216±0.0021
9	0.8842±0.0015	0.9204±0.0022
10	0.8887±0.0018	0.9227±0.0018
Number of clusters = 15		
No. of Runs	AUROC	AUPRC
1	0.8934±0.0015	0.9300±0.0011
2	0.8923±0.0008	0.9253±0.0013
3	0.8891±0.0019	0.9225±0.0014
4	0.8905±0.0016	0.9266±0.0009
5	0.8868±0.0027	0.9201±0.0031
6	0.8942±0.0019	0.9303±0.0012
7	0.8923±0.0005	0.9260±0.0014
8	0.8937±0.0020	0.9284±0.0022
9	0.8923±0.0021	0.9273±0.0007
10	0.8939±0.0019	0.9279±0.0026
Number of clusters = 20		
No. of Runs	AUROC	AUPRC
1	0.8916±0.0016	0.9274±0.0015
2	0..8851±0.0016	0.9220±0.0021
3	0.8827±0.0017	0.9182±0.0014
4	0.8912±0.0007	0.9266±0.0011
5	0.8923±0.0006	0.9285±0.0010
6	0.8872±0.0012	0.9242±0.0006
7	0.8815±0.0014	0.9214±0.0024
8	0.8864±0.0009	0.9224±0.0014
9	0.8929±0.0027	0.9317±0.0022
10	0.8886±0.0019	0.9259±0.0015

9. Cluster-based train-test tasks for meta-learning

We also evaluated the performance of meta-learning method on more challenging cluster-based splits. A leave-one-cluster-out (LOCO) approach where one cluster is kept as test task, while

remaining clusters are used as training tasks is considered. With a total of 10 clusters (Table S6), we use one cluster as test task and other 9 clusters as training tasks. This results in a combination of 10 different train-test tasks. Three different support set sizes are considered: 16, 32, and 64. The query set size is chosen to be 128. The approach followed for meta-testing is same as shown in Figure 5c. The support and query sets are part of the test task. The model performance averaged over ten different support-query random splits of every test task is reported in Table S16.

Table S16. Model Performance of Prototypical Networks in Terms of AUROC and AUPRC Scores with Different Clusters in Training and Test Tasks

Cluster C1		
Support set size	AUROC	AUPRC
16	0.7319±0.0536	0.7906±0.0481
32	0.7393±0.0667	0.8058±0.0685
64	0.8014±0.0466	0.8595±0.0511
Cluster C2		
Support set size	AUROC	AUPRC
16	0.6486±0.0461	0.6743±0.0411
32	0.6530±0.0326	0.6829±0.0518
64	0.6729±0.0646	0.7123±0.0770
Cluster C3		
Support set size	AUROC	AUPRC
16	0.6949±0.0879	0.8308±0.0509
32	0.7277±0.0247	0.8392±0.0381
64	0.7480±0.0440	0.8624±0.0330
Cluster C4		
Support set size	AUROC	AUPRC
16	0.6815±0.0736	0.7928±0.0672
32	0.6978±0.0310	0.8275±0.0445
64	0.7291±0.0477	0.8362±0.0559
Cluster C5		
Support set size	AUROC	AUPRC
16	0.6757±0.0933	0.7586±0.0733
32	0.7286±0.0583	0.8084±0.0376
64	0.7750±0.0368	0.8415±0.0335
Cluster C6		

Support set size	AUROC	AUPRC
16	0.6548±0.0658	0.8004±0.0360
32	0.6798±0.0553	0.8215±0.0371
64	0.7574±0.0341	0.8744±0.0285
Cluster C7		
Support set size	AUROC	AUPRC
16	0.5929±0.0671	0.7625±0.0535
32	0.6316±0.0510	0.7858±0.0325
64	0.6973±0.0843	0.8155±0.0621
Cluster C8		
Support set size	AUROC	AUPRC
16	0.6890±0.0691	0.8622±0.0450
32	0.7475±0.0720	0.8856±0.0426
64	0.7890±0.0460	0.9062±0.0277
Cluster C9		
Support set size	AUROC	AUPRC
16	0.6458±0.0674	0.7090±0.0643
32	0.6825±0.0596	0.7497±0.0478
64	0.7461±0.0419	0.7846±0.0365
Cluster C10		
Support set size	AUROC	AUPRC
16	0.6269±0.1173	0.8588±0.0470
32	0.7020±0.0556	0.8713±0.0528
64	0.7633±0.0538	0.9072±0.0353

A comparison of model performance of meta-learning with single-task methods on cluster-based splits is provided in Table S17. The results are reported for single-task methods trained on all training clusters.

Table S17. Model Performance of RF and GNN in Terms of AUROC and AUPRC Scores with Different Clusters in Training and Test Tasks

	RF		GNN	
	AUROC	AUPRC	AUROC	AUPRC
Cluster C1	0.7136±0.0285	0.7145±0.0275	0.6942±0.0159	0.7044±0.0133
Cluster C2	0.6583±0.0364	0.6269±0.0332	0.6298±0.0028	0.6106±0.0020
Cluster C3	0.6667±0.0276	0.7669±0.0240	0.6414±0.0040	0.7388±0.0022
Cluster C4	0.6881±0.0400	0.7472±0.0409	0.6309±0.0080	0.7202±0.0047
Cluster C5	0.6751±0.0484	0.7292±0.0439	0.6442±0.0075	0.7128±0.0045
Cluster C6	0.6778±0.0441	0.7829±0.0537	0.6836±0.0066	0.7779±0.0038
Cluster C7	0.6172±0.0379	0.7401±0.0436	0.6140±0.0041	0.7325±0.0022

Cluster C8	0.7327±0.0452	0.8572±0.0346	0.6980±0.0066	0.8259±0.0030
Cluster C9	0.6187±0.0275	0.6394±0.0329	0.6476±0.0091	0.6653±0.0065
Cluster C10	0.6822±0.0323	0.8338±0.0228	0.6428±0.0091	0.8288±0.0037

10. Performance on out-of-sample test set

To demonstrate the generalizability of our meta-models, we analyzed the performance on an out-of-sample test set. The results are reported in terms of AUPRC and AUROC scores as an average over 10 random support-query splits. Table S18 presents the results with the standard meta-learning framework at meta-test time. It can be noted that the meta-learning method consistently perform better than the single-task methods.

Table S18. Model Performance of Meta-learning and Single-task Methods on Out-of-sample Test Set Using Standard Meta-Learning Framework

	Prototypical network	
Support set size	AUPRC	AUROC
8	0.5952 +- 0.0258	0.8676 +- 0.0143
16	0.6554 +- 0.0259	0.8672 +- 0.0219
32	0.7386 +- 0.0250	0.9133 +- 0.0232
64	0.7984 +- 0.0127	0.9341 +- 0.0053
Full training data	0.7141 +- 0.0128	0.9286 +- 0.0062
	RF	
Support set size	AUPRC	AUROC
8	0.5380 +- 0.0152	0.8100 +- 0.0123
16	0.5418 +- 0.0174	0.8132 +- 0.0090
32	0.5934 +- 0.0145	0.8501 +- 0.0054
64	0.6353 +- 0.0087	0.8631 +- 0.0061
Full training data	0.6345 +- 0.0169	0.8728 +- 0.0041
	GNNs	
Support set size	AUPRC	AUROC
8	0.5189 +- 0.0145	0.8148 +- 0.0115
16	0.5696 +- 0.0121	0.8396 +- 0.0070
32	0.5840 +- 0.0390	0.8356 +- 0.0169
64	0.7147 +- 0.0170	0.8764 +- 0.0090
Full training data	0.6395 +- 0.0115	0.8558 +- 0.0042

Table S19 presents the results with the meta-cluster approach at meta-test time. It can be noted that the meta-learning method consistently perform better than the single-task methods. The standard meta-learning and meta-cluster provides comparable performance.

Table S19. Model Performance of Meta-learning and Single-task Methods on Out-of-sample Test Set Using Meta-Cluster Approach

	Prototypical network	
Support set size	AUPRC	AUROC
8	0.6232 +- 0.0472	0.8881 +- 0.0150
16	0.6147 +- 0.0271	0.8875 +- 0.0112
32	0.6484 +- 0.0103	0.8972 +- 0.0052
64	0.6633 +- 0.0083	0.9015 +- 0.0028
Full training data	0.6939 +- 0.0053	0.9147 +- 0.0022
	RF	
Support set size	AUPRC	AUROC
8	0.4922 +- 0.0048	0.8339 +- 0.0023
16	0.4969 +- 0.0060	0.8377 +- 0.0024
32	0.5170 +- 0.0043	0.8528 +- 0.0036
64	0.5245 +- 0.0131	0.8485 +- 0.0031
Full training data	0.5880 +- 0.0054	0.8692 +- 0.0017
	GNNs	
Support set size	AUPRC	AUROC
8	0.4884 +- 0.0281	0.8350 +- 0.0088
16	0.5435 +- 0.0280	0.8544 +- 0.0101
32	0.5190 +- 0.0103	0.8459 +- 0.0045
64	0.4422 +- 0.0344	0.8218 +- 0.0071
Full training data	0.4825 +- 0.0240	0.8021 +- 0.0072
